# MiCASA is a new method for quantifying cellular organization

**DOI:** 10.1038/ncomms15619

**Published:** 2017-05-30

**Authors:** Andrew Sornborger, Jie Li, Cullen Timmons, Floria Lupu, Jonathan Eggenschwiler, Yousuke Takahama, Nancy R. Manley

**Affiliations:** 1 Department of Mathematics, University of California, Davis, California 95616, USA; 2 Department of Genetics, Paul D. Coverdell Center, University of Georgia, 500 DW Brooks Drive, Athens, Georgia 30602, USA; 3 Division of Experimental Immunology, Institute for Genome Research, University of Tokushima, Tokushima 770-8503, Japan

## Abstract

While many tools exist for identifying and quantifying individual cell types, few methods are available to assess the relationships between cell types in organs and tissues and how these relationships change during aging or disease states. We present a quantitative method for evaluating cellular organization, using the mouse thymus as a test organ. The thymus is the primary lymphoid organ responsible for generating T cells in vertebrates, and its proper structure and organization is essential for optimal function. Our method, Multitaper Circularly Averaged Spectral Analysis (MiCASA), identifies differences in the tissue-level organization with high sensitivity, including defining a novel type of phenotype by measuring variability as a specific parameter. MiCASA provides a novel and easily implemented quantitative tool for assessing cellular organization.

## Abstract (web summary)

There are few methods available that can quantify relationships between cell types in tissue images. Here the authors present a quantitative method to evaluate cellular organization, validated in the mouse thymus and spinal cord, called Multitaper Circularly Averaged Spectral Analysis (MiCASA).

The lack of quantitative methods to assess and describe the organization, as opposed to composition, of tissues and organs is a significant technical and theoretical barrier in the study of many organs and tissues. Organs are more than the sum of their component parts—functional competence requires that these parts not only be present in the appropriate proportions, but also be arranged in specific ways. However, there are few quantitative tools for evaluating and comparing tissue organization. As a result, organization is usually assessed by qualitative and subjective methods (whether it ‘looks organized'). This lack of tools constitutes a critical problem; without a quantitative framework to characterize the functional organization of organs and tissues, we currently do not have a language to describe its disintegration during disease or involution, or benchmarks to evaluate regenerative therapies.

The thymus is an excellent example of the connection between organization and function. The thymus consists of developing T cells, or thymocytes, supported by a complex cellular environment containing a variety of resident cell types, including thymic epithelial cells (TEC), dendritic cells, vasculature and mesenchymal cells^1^. These cell types comprise multiple microenvironments that direct and support thymocytes to develop from immature progenitors into mature T cells that are both self-tolerant (will not attack the body's own cells) and self-restricted (only recognize antigens in a specific ‘self' context). T-cell development in the thymus is not a cell autonomous process, but requires interactions with the thymic microenvironments that provide signals for their survival, proliferation and differentiation^2^,^3^,^4^,^5^. Failure of these events results in immunodeficiency or autoimmunity.

Thymic output is quantitatively and qualitatively correlated with peripheral immune function. Loss of thymic output occurs during aging and because of a wide variety of conditions including genetic disorders, disease and cancer therapies such as irradiation and chemotherapy^6^,^7^. Transient or permanent thymic rejuvenation thus has major consequences for human health.

Establishing quantitative, predictive models of thymic structure and function could have significant implications for understanding the process of immunosenescence and for evaluating the effectiveness of clinical interventions. In spite of its critical role in the generation of cellular immunity, the composition and organization of thymic microenvironments and the mechanisms that promote proper development and function are not fully understood. To date, no quantitative models of thymus organ structure and function exist in the literature. In this report, we have addressed this need by developing a statistical framework for measuring cellular associations that quantify cellular organization in an organ or tissue, in this case the mouse postnatal thymus. We then use this tool to evaluate organ structure in previously published mutant strains with defects in TEC differentiation and organ structure. These analyses show that Multitaper Circularly Averaged Spectrum Analysis (MiCASA) can detect statistically robust phenotypic differences at earlier stages than can be identified by the eye, and can identify novel types of phenotypic differences including changes in the variability (Variance) of cellular organization. We also show MiCASA analysis of two different sets of markers in wild-type mouse fetal spinal cord, demonstrating that the resulting methodology may be used, more generally, for characterizing cellular organization elsewhere, including tissues outside of the immune system.

## Results

### Calculating relative spatial distributions of cell types

To develop both a rapid screening method for assessing thymic organization and a quantitative method to assess specific cellular associations within the thymus, we developed a statistical framework for measuring cellular associations based on cellular correlation functions. We calculate these correlation functions in the frequency domain and average them over all angles, providing a summary of the distribution of individual cell types and associations between cell types. We call our method MiCASA. Similar analytical methods are commonly used in cosmology to describe and quantify structure in the distribution of galaxies throughout the universe^8^,^9^,^10^. In the resulting graphs, the ordinate measures structure within a cellular distribution (log-spectra) or correspondences between two distinct cell distributions (atanh-coherence). The abscissa represents the logarithm of spatial frequency, which, in turn, is inversely proportional to the intercellular distance. Thus, this axis essentially measures characteristic cellular separations, with distances decreasing to the right of the graph. Each parameter can be evaluated for statistical significance, giving a quantitative and sensitive measure of cellular organization. Because of the ability of each parameter's distribution to be analysed for statistical significance, our approach can yield a great deal of detailed information about not only whether a sample is organized, but also in what way its organization differs from wild type.

**Figure 1:**
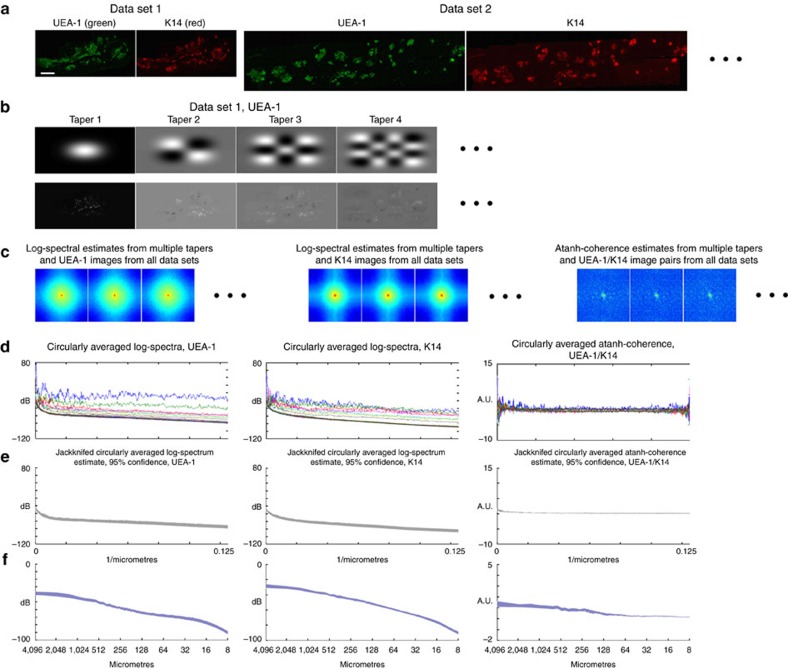
Intermediate steps in a MiCASA analysis. (**a**) Representative images of 1-month-old male thymus from a set of fluorescence imaging experiments measuring the same condition. (**b**, top). The first four Slepian tapers used in the analysis. A similar set (but with differing pixel number to accommodate the different sizes of images from the experiment) of M tapers with the same bandwidth, *W*, multiply all fluorescence images from all N experiments, giving a set of *N* times M tapered estimates (**b**, bottom). (**c**) Two-dimensional log-spectra for UEA-1 and K14 (first and second sets of panels) and atanh-coherence (third set of panels) are calculated for all *NM* tapers and experiments. (**d**) The sets of log-spectra (first two panels) and atanh-coherence are interpolated to the same-frequency grid and circularly averaged, providing information about the cellular distribution at different spatial frequencies. (**e**) Jackknife confidence intervals are calculated from the sets of log-spectra and coherence, giving the final 99% confidence intervals for the log-spectra and atanh-coherence. Note that by plotting confidence intervals for two conditions, one may do statistics by eye, that is, if the confidence intervals do not overlap in a given frequency band, then there is at least 99% confidence that the conditions differed. (**f**) Jackknife confidence intervals for the log-spectra and coherence shown in (**e**), but plotted as a function of log-frequency and labelled with the length scale of each frequency in micrometres. Scale bar (**a**), 100 μm.

The method is technically straightforward. First, we prepare the samples using fluorescent immunohistochemistry to detect two different cell types on sagittal sections taken from the central third of the thymus of age- and sex-matched control and experimental animals. Each entire stained section is then photographed at high magnification (generally using a × 40 objective) using a tiled pattern, such that each image overlaps the adjacent ones. The tiled images are compiled into a single high-resolution image (typically 2 μm per pixel), either manually or using a software programme such as PTGui (Panoramic Tools Graphical user interface). The correlation analysis is coded in MATLAB. The analytical approach is demonstrated in Fig. 1.

To quantify the co-localization of two cell types, we first calculated two-dimensional Fourier transforms of these tiled high-resolution images from a minimum of three individuals with the same genotype. The data in Fig. 1 use two data sets from wild-type thymi generated from different axial levels within the organ, resulting in sections of differing sizes but with similar cellular organization (Fig. 1a). The data are then multiplied by a set of Slepian tapers (Fig. 1b) and Fourier-transformed. The logarithm of the squared amplitudes of the tapered Fourier coefficients (log-spectra; Fig. 1c, two left panels) and the arc-hyperbolic-tangent of the normalized tapered cross-spectrum (atanh-coherence; Fig. 1c, right panel) are then circularly averaged to obtain a measure of cell clustering at different spatial frequencies (log-spectra; Fig. 1d, two left panels), and a measure of co-localization of cluster types (atanh-coherence; Fig. 1d, right panel). Confidence intervals (CIs) are then computed for the log-spectra and atanh-coherences (Fig. 1e). Finally, coordinates on the abscissa are converted to logarithmic coordinates and labelled with the spatial scales corresponding to inverse spatial frequencies. This last step allows the user, at a glance, to see the spatial scale of the wavelength that a feature occurs at. Multitaper methods avoid some typical pitfalls of Fourier transforms on random data and have most commonly been used for the analysis of time series^11^,^12^. Here we extend their use to two-dimensional Fourier transforms. From a practical standpoint, multitaper methods allow us to calculate CIs on moments of the Fourier transform, even on a single data set (Fig. 1c,d). For a given analysis, *N* images are processed using *M* tapers, giving *NM* degrees of freedom. The number of tapers, *M*, may be thought of loosely as the number of sections a given image is divided into for the analysis. *M* is proportional to a smoothing parameter, *W* (see Supplementary Note), and may be increased by making this parameter larger. This has the effect of smoothing the estimates across a wider band of frequencies. In this paper, we choose *W* such that *M*=7. The user will have to adjust *W* (and hence *M*) for their own purposes (see Supplementary Note). For this value, we get good frequency resolution (not too much smoothing), but still have sufficient degrees of freedom for a stable estimate. CIs are computed based on standard *t*- or *χ*^2^-tests, and thus the results may be interpreted in the usual way. A complete pedagogical description of the method and the reasoning behind its design are given in the Supplementary Note.

A typical analysis is performed on paraffin or frozen sections of a tissue (in this case a thymus) that have been fluorescently labelled for two markers that identify nonoverlapping cell subsets (either by immunohistochemistry or with genetic fluorescent-tagging techniques). The MiCASA analysis results in three primary output functions with CIs, the circularly averaged log-spectra of the first and second markers and the circularly averaged atanh-coherence (Fig. 1e,f). In addition, we compute estimates of the variances of these three output functions. The CIs allow the user to distinguish multiple specific phenotypes based on the distribution of log-spectra or atanh-coherences represented by the input images. The log-spectrum of the individual markers identifies differences in the distributions of each cell type across the tissue section. The atanh-coherence identifies differences in the organization of the two cell types relative to each other, and is thus a measure of the compartmental organization of the thymus. The variances that we compute allow us to quantify the consistency of organization within or between conditions. For all results in this paper, *M*=7, *N* is reported in the figure legend for each analysis, and in each case, the width of the line on the resulting graphs represents the 99% CI, and separations between CIs are indications of significance.

As a test of the consistency of outputs generated using MiCASA, we compared sections from two different 1-month-old male wild-type thymi, sectioned, processed, stained and imaged in parallel. The graphs for the individual log-spectra and atanh-coherence were completely overlapping at the 99% CI (Supplementary Fig. 1).

### Validation in a genetic model of early thymus involution

We compared control and mutant mice labelled either for cortical and medullary markers or for two nonoverlapping medullary cell types to demonstrate how MiCASA may be used to provide a useful summary of the distribution and interactions between well-known cell types in the thymus, and how these parameters are changed in specific mutants. This analysis identified several previously unidentified statistically significant differences in the log-spectra and atanh-coherence of both cortical and medullary markers. These differences were distributed across many length scales and the MiCASA results were found to be a useful summary of a number of differences in the cellular distribution.

*Foxn1*^*Z/Z*^ mutants develop normally, but have a postnatal reduction in expression of the key TEC transcription factor FOXN1 beginning at ∼2 weeks of age. This downregulation results in rapid and progressive changes in both thymus size and apparent corticomedullary organization that mirror those seen during aging-related thymic involution^13^. We evaluated the phenotypes of *Foxn1*^*Z/Z*^ mutants by comparing them to *Foxn1*^*+/Z*^ littermate controls using MiCASA.

**Figure 2:**
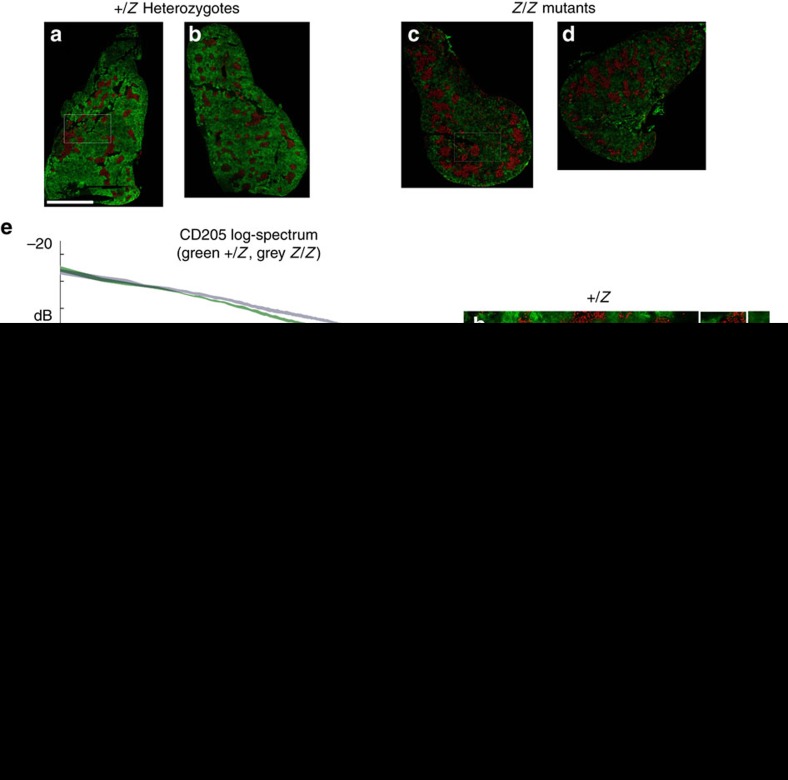
Analysis of the distribution of cortical and medullary cell populations in control and *Foxn1*^*Z/Z*^ mutant thymus at 4 weeks. The cortical marker (CD205) is green and the medullary marker (K14) is red. (**a**–**d**) Representative images of *Foxn1*^*+/Z*^ control (**a**,**b**; from three sections each of *N*=3 thymi with M=7 tapers, giving total degrees of freedom, *NM*=21) and *Foxn1*^*Z/Z*^ mutant (**c**,**d**; *N*=3 thymi, with M=7 tapers, giving total degrees of freedom, *NM*=21) thymi reconstructed from multiple high-resolution images. (**e**–**g**) Graphical outputs from MiCASA: the log-spectra for the cortical (**e**) and medullary (**f**) markers, and the atanh-coherence (**g**). (**h**–**k**) Examples of structures on intermediate (**h**,**j**; 50–400 μm) and fine (**i**,**k**; 8–24 μm) scales for both control and mutant as indicated. All MiCASA results are plotted as 99% confidence bands. Scale bars (**a**–**d**), 2,750 μm; (**h**,**j**), 400 μm; (**i**,**k**), 100 μm.

Figure 2 shows an analysis of CD205 (cortical, green fluorescence) and K14 (medullary, red fluorescence) expression in 1-month-old male heterozygous control (Fig. 2a,b) and mutant (Fig. 2c,d) thymi. At this stage, differences in thymic cellular organization are easily identifiable, and thus this analysis should detect multiple significant differences between the mutants and controls. The CD205 log-spectrum (Fig. 2e) for both control (green) and mutant (grey) are superimposed showing a statistically significant difference (*P*>>99% increase in power) in the cortical cell distribution in a wide band (160–15 μm) around the 40 μm scale (spatial frequency of 1/40 μm^−1^). This corresponds to a change from a smooth distribution of green fluorescence in the images to a dappled pattern at these length scales in mutants (compare Fig. 2h,j). The K14 marker's log-spectrum (Fig. 2f) for control (red) and mutant (grey) show statistically significant differences in a small band about the 80 μm scale (*P*>>99% increase in power) and a large band about the 12 μm scale (*P*>>99% decrease in power). The increase in power at 80 μm is due to the increase in size of the medullary islets in mutants. The decrease in power in the band ∼12 μm corresponds to an increase in cell-to-cell distance within the medullary islets (Fig. 2i,k) in the mutant relative to control. Thus, power at high frequencies decreased. The peaks in the atanh-coherence in both controls and mutants that were centred at the 400 μm scale were due to a prominent anticorrelation of cortical and medullary tissue. In mutants, the peak decreased in amplitude and the peak centre shifted to lower spatial frequency (longer length scale), indicating an increased intermingling of cortical and medullary markers and larger medullary islets. Taken together, this analysis shows changes in cell distribution within both the cortical and medullary domains. In the control, cortical and medullary markers exist in distinct regions from each other, but in the mutant, the regional differentiation is less distinct (lower amplitude of atanh-coherence) and the islets are of larger size (spatial frequency of peak shifted to lower frequency; compare islets in A,B with C,D). These differences are characteristic of changes seen with aging-related thymic involution.

**Figure 3:**
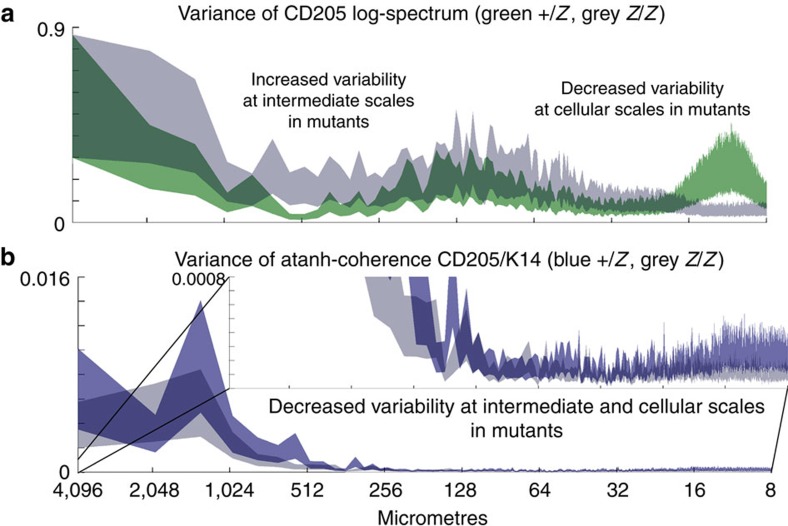
Analysis of variance in corticomedullary organization in heterozygote control and *Foxn1*^*Z/Z*^ mutant thymus at 4 weeks. The *y-*axis represents the variance of the atanh-coherence, and the width of the lines in these graphs represents 99% confidence intervals on this quantity. Graphs in this figure were derived from the same data sets as those in Fig. 2. (**a**) Variance in the lag-spectrum of CD205+ cell distributions in *Foxn1*^*+/lacZ*^ controls compared to *Foxn1*^*lacZ/lacZ*^ mutant at 4 weeks of age. (**b**) Variance in the CD205/K14 atanh-coherence. Inset shows an expansion of the *y-*axis to highlight the significant differences (gaps between the control and mutant graphs) seen at this scale. All MiCASA results are plotted as 99% confidence bands.

In addition to measures of the log-spectrum and atanh-coherence, it is important to assess their variance. Variance is a measure of the consistency of phenotypes within a condition. It is common in mutants for phenotypes not only to change, but to become more variable. With MiCASA, changes in phenotype are measured with the log-spectra and atanh-coherence, whereas phenotypic consistency is measured by their respective variances. In the case of cellular organization, this distinction is between cells assuming a different organization and becoming disorganized. The variances of the CD205 log-spectrum and CD205/K14 atanh-coherence are given in Fig. 3a,b. While the phenotypic differences shown in the CD205 log-spectrum were centred around the 40 μm scale. Surprisingly, the variability of the log-spectrum decreased in *Z*/*Z* mutants at cellular and nearest neighbour scales. In contrast, variance of the atanh-coherence increased significantly at essentially all length scales. The most significant difference was at the largest scales (Fig. 3b, inset). This is an indication that, for this mutant phenotype, there is a widespread disruption in corticomedullary compartmentalization.

**Figure 4:**
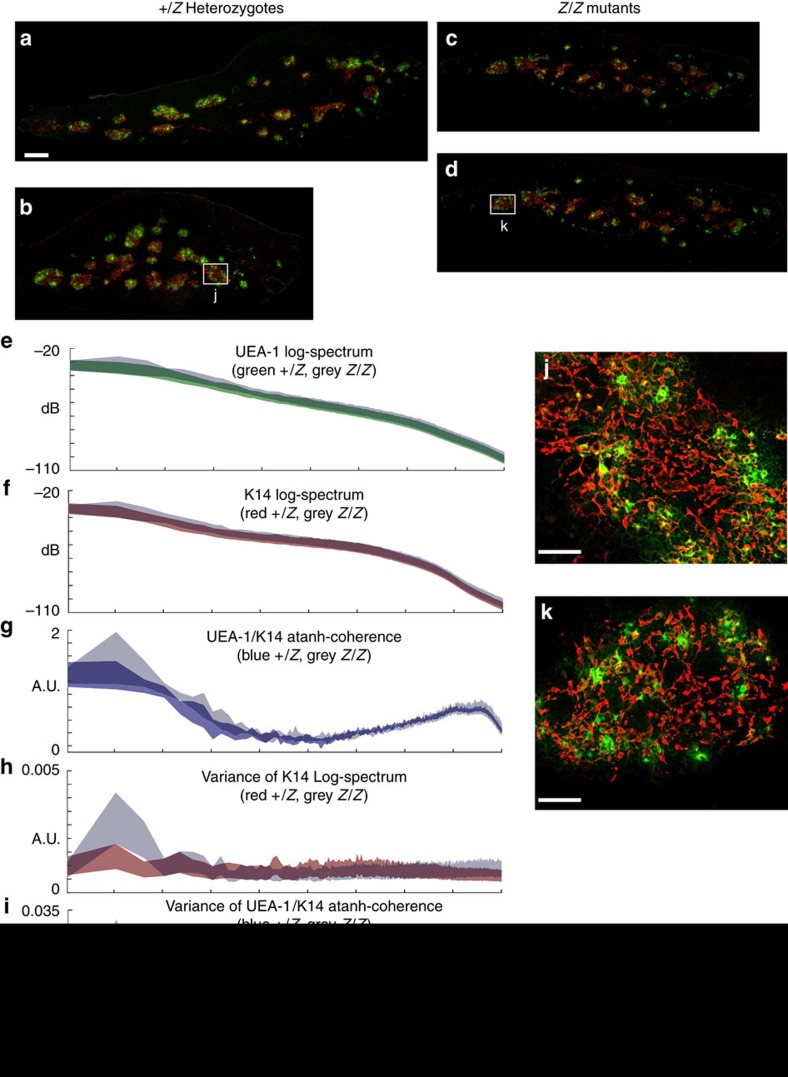
Analysis of medullary organization in the thymus of *Foxn1*^*Z/Z*^ mutants at 2 weeks. (**a**–**d**) Representative images of *Foxn1*^*+/Z*^ control (**a**,**b**; from *N*=4 thymi, with *M*=7, giving total degrees of freedom, *NM*=28) and *Foxn1*^*Z/Z*^ mutant (**c**,**d**; from *N*=4, with *M*=7, giving total degrees of freedom, *NM*=28) thymi reconstructed from multiple high-resolution images. The first medullary marker (UEA-1) is green and the second medullary marker (K14) is red. (**e**–**i**) Graphical outputs from MiCASA: the log-spectra for the UEA-1 (**e**) and K14 (**f**) markers, the atanh-coherence (**g**), and Variance plots for the K14 log-spectrum (**h**) and the atanh-coherence (**i**). (**j**,**k**) Examples of structures on intermediate (50–400 μm) scales for control (**j**) and mutant (**k**); locations of these high-resolution images are shown in boxes in **b**,**d**. All MiCASA results are plotted as 99% confidence bands. Scale bars (**a**–**d**), 1,180 μm; (**j**,**k**), 180 μm.

To determine the sensitivity and detection threshold of our method, we assessed organization of these mutants immediately after *Foxn1* downregulation, at 2 weeks of age, when phenotypes are first developing, but prior to any identified differences in stromal organization. Figure 4 shows MiCASA results for two nonoverlapping medullary markers, K14 and UEA-1. Figure 4a–d shows representative fluorescent images used in the analysis. Neither marker showed any statistically significant differences in their log-spectra (Fig. 4e,f). There was one very small gap in Atahn-coherence centred ∼800 μm, indicating a small statistically significant difference in organization at a scale similar to between medullary islet distances (Fig. 4g). In addition, there was a difference in the variance of the K14 log-spectrum centred around ∼2,000 μm (Fig. 4h), and multiple large and statistically significant differences between the variance of the atanh-coherences across the entire length scale (Fig. 4i). The differences at large scale indicate that overall medullary organization at the organ-wide scale was disrupted, although this difference is not obvious to the eye (Fig. 4a–d). The fine-scale differences from 8 to 16 μm scales indicate that there were large inconsistencies in the relative locations of individual cells and how cells interacted with their neighbours (Fig. 4i inset). This difference is reflected in the looser and more variable organization of cells within medullary islets (Fig. 4i–k). Thus, MiCASA detected subtle changes in the organization of the thymus early after downregulation, with the earliest changes involving an increased variability in both the organization of medullary islets across the organ as well as in local arrangements of these two cell types relative to each other within the medullary islets.

### Validation in a genetic model affecting thymus organization

**Figure 5:**
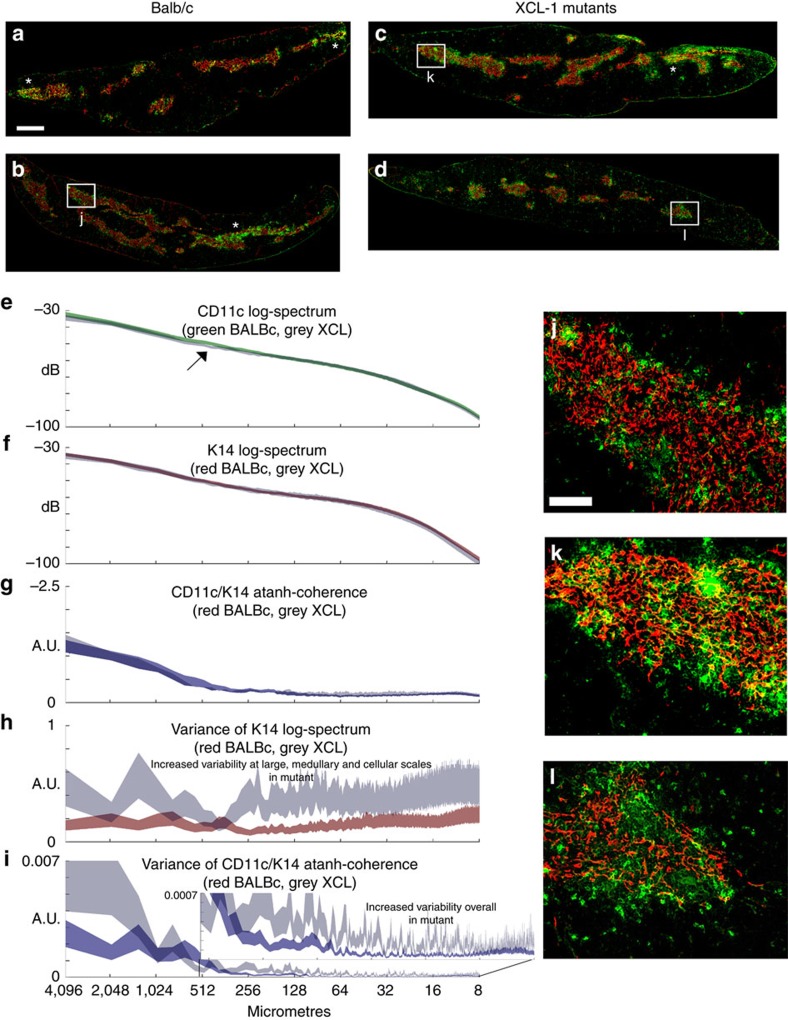
Analysis of CD11c distribution relative to the medulla in *Xcl1* mutants. (**a**–**d**) Representative images of 6-week-old male Balb/c wild-type control (**a**,**b**; from *N*=5 thymi, with *M*=7, giving total degrees of freedom, *NM*=35) and *Xcl1*^*−/−*^ mutant (**c**,**d**; from *N*=4 thymi, with *M*=7, giving total degrees of freedom, *NM*=28) thymi reconstructed from multiple high-resolution images. The dendritic cell marker (CD11c) is green and the medullary marker (K14) is red. Clustering of the CD11c marker within the medulla is indicated by asterisks (*). (**e**–**i**) Graphical outputs from MiCASA: the log-spectra for the CD11c (**c**) and K14 (**d**) markers, the atanh-coherence (**e**) and the Variance plots for the K14 log-spectrum (**f**) and the atanh-coherence (**g**). Arrow in **c** indicates a single significant difference between the control and mutant log-spectra for CD11c. All MiCASA results are plotted as 99% confidence bands. (**j**–**l**) Examples of structures on intermediate (50–400 μm) scales, identified as boxed regions in **b**–**d**. Scale bars (**a**–**d**), 1,230 μm; (**j**,**k**), 220 μm.

The analysis of the marker pairs shown above can be used as a generic tool for analysing corticomedullary organization. However, MiCASA can be used with any pair of markers. Therefore, we re-analysed the phenotype of the XCL1 mutants previously reported by the Takahama lab. These mutants were reported to have a mislocalization of CD11c+ dendritic cells out of the medulla and into the cortex^14^. We performed MiCASA on 6-week-old adult male thymi using CD11c and K14 markers to investigate this phenotype and to test whether additional phenotypes could be detected by this method (Fig. 5). Upon generation of the reconstructed high-resolution images, it was immediately obvious that, in both controls and mutants, there was section-to-section variability in the distribution of CD11c+ cells across the medulla, with some sections showing large accumulations of CD11c+ cells in different locations within the medulla (Fig. 5a–d, asterisks), while other regions had relatively fewer CD11c+ cells. We did note regions with significant accumulations of DCs outside of the medullary regions in the mutants, consistent with the previously described phenotype (Fig. 5l)^14^. However, there are also DCs just outside the medulla in controls in some places, and regions in the mutants where this displacement was less obvious relative to controls (compare Fig. 5j–l). Therefore, DC localization in both the controls and mutants is complex, and the nature and origins of any defects are not necessarily obvious. As an independent measure of DC localization, we performed region-specific cell counts on the whole reconstructed sections used for MiCASA, using CellProfiler. This analysis did confirm a significant increase in DCs outside the medulla, particularly in the subcapsular cortical region (Supplementary Fig. 2). However, there was also high variability identified in these whole-section cell counts, consistent with the appearance of the reconstructed section images.

On the basis of the reported phenotype, we expected MiCASA to find a significant difference in the atanh-coherence, reflecting a difference in the relative organization of CD11c+ cells to the medulla. We found no difference between the mutants and controls in either the individual K14 log-spectrum or the atanh-coherence (Fig. 5e–g). There was a small specific difference in the CD11c log-spectrum at the 500 μm scale (Fig. 5e, arrow). As this scale correlates to the width of medullary regions, this difference could easily reflect the previously reported displacement of DCs out of the medulla into the cortico-medullary junction (CMJ). Remarkably, there were clearly significant differences in the variance of both the K14 log-spectrum (Fig. 5h) and the CD11c/K14 atanh-coherence (Fig. 5i). In both cases, the mutants were consistently both more variable (wider line) and had more cells that were more variable (grey line above the other line) at multiple length scales. This result indicates a widespread disruption in the consistency of both within- and between-medullary organization (K14) and in the localization of CD11c relative to the medullary regions (atanh-coherence). This increase in variance is the more dramatic phenotype, and could both represent a novel phenotype in itself and be the underlying cause of the mislocalization previously reported.

### Evaluating cellular organization of the fetal spinal cord

**Figure 6:**
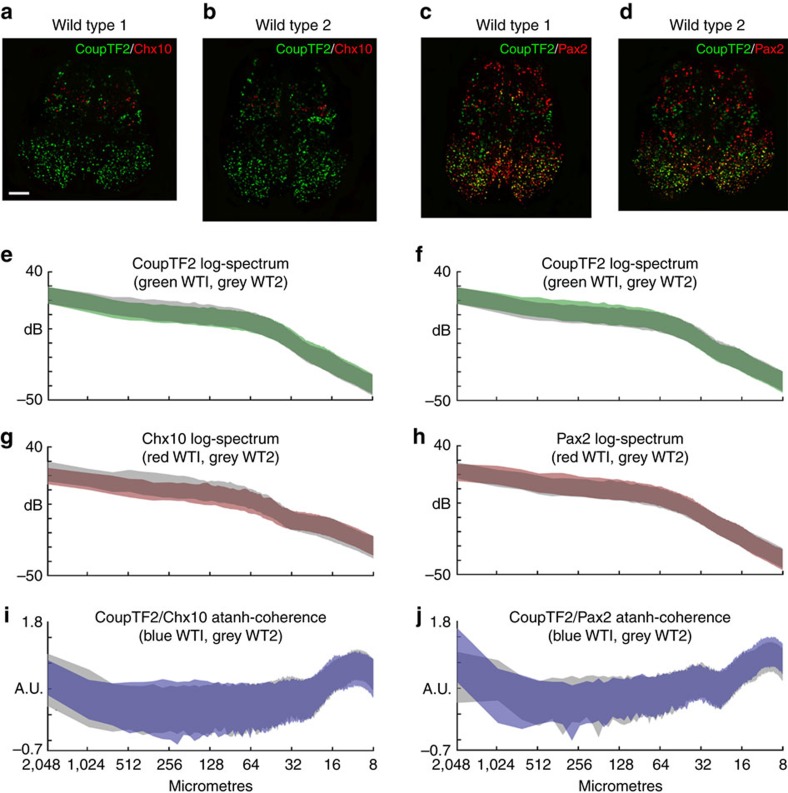
Analysis of fetal spinal cord organization using MiCASA. (**a**–**d**) Representative images of transverse sections from two different E15.5 fetal spinal cord samples (wild type 1 and 2) stained with either CoupTF2 and Chx10 (**a**,**b**) or CoupTF2 and Pax2 (**c**,**d**). Images were reconstructed from multiple high-resolution images. *N*=5 thymi, with *M*=7, giving total degrees of freedom, *NM*=35 for each sample and staining combination. For each pair of markers, the two wild-type samples were compared to each other using MiCASA. Individual log-spectra (**e**–**h**) and atanh-coherence (**i**,**j**) are shown. No significant differences were seen between the samples. All MiCASA results are plotted as 99% confidence bands. Scale bar, 100 μm.

The MiCASA method relies on the measurement of simple parameters (distances between fluorescently labelled cells in an image) to characterize cellular and tissue organization. As such, it should be applicable to any sample in which elements can be labelled in this way, regardless of the tissue type or specific markers of interest. To test the broad applicability of this approach, we performed MiCASA on images of E15.5 fetal mouse spinal cord stained with three markers that label different regions, CoupTF2, Pax2 and Chx10; for MiCASA, we compared these three markers in two pairwise combinations (Fig. 6). For this analysis, we compared samples from two wild-type embryos to each other; therefore, the general shape and overall patterns should be similar, although the exact dimensions of the tissue would differ slightly due to small differences in tissues after fixation and processing.

The log-spectra of the individual markers for both comparisons were completely overlapping, indicating that each of the wild-type samples had similar MiCASA outputs (Fig. 6e–h). The general shapes of the log-spectra were also similar, and similar in general to those seen from the adult thymus samples (higher to the left and trending downward toward the right). Given the differences in the thymus and spinal cord samples, this likely reflects a general principle of the way MiCASA characterizes tissues, for example, there will usually be fewer cells closer to a reference point and more cells farther away. The atanh-coherence graphs for the two sets of spinal cord markers were also very similar to each other (Fig. 6i,j), but in this case were quite different from the thymus results in Figs 2g and 4g. As this measure reflects the correlations between the two markers, this measure is more likely to reflect tissue-specific properties of cellular organization.

## Discussion

The real utility and power of the MiCASA method is that it is an unbiased, user-independent method for identifying the phenotypic differences between conditions. Therefore, it focuses your attention on those aspects of the phenotype that have statistical relevance. That is, rather than identifying possible changes by eye and then trying to find a statistical method to test whether those apparent changes are ‘real', statistically significant differences in both phenotype and variance can be identified first, and then investigated in more detail. MiCASA thus is not only a useful new tool, but represents a complete change in the way that tissue structure and cellular organization phenotypes are approached experimentally and analytically.

The analyses of the two mutants in this study highlight the utility of the MiCASA method for assessing phenotypes. In both cases, the mutants have been previously published by one of our labs, reporting specific phenotypes. The Manley lab previously reported that the earliest changes in stromal organization in the *Foxn1*^*Z/Z*^ mutant were reliably detected at 3 weeks^13^. The MiCASA method is able to detect changes in cellular organization a full week earlier, within a week of when molecular changes initiate. The main initial changes were in the variance of the atanh-coherence, indicating that the first cellular event we detect is a breakdown in the organization of cellular relationships within the medulla. MiCASA is thus a more sensitive tool than visual comparison for identifying differences in phenotype, and can detect a change that is very difficult to detect by visual inspection.

The re-analysis of the previously published XCL1 mutants provides a different example, in which a previously identified phenotype is viewed in a new light, suggesting an additional phenotype that could underlie or contribute to the differences originally identified. The original report of these mutants concluded that in mice deficient for XCL1, thymic dendritic cells (tDCs) failed to efficiently accumulate CD11c+ tDCs in the medulla^14^. MiCASA did detect a very specific difference at a narrow length distance that could correlate with this reported phenotype. However, MiCASA also detected a more general disruption of medullary structure (based on increased variance in K14+ cell organization in mutants) and in dendritic cell localization within the medulla (based on increased variance of CD11c/K14 correlation in the atanh-coherence). These increases in variance occurred across a broad range of cellular scales, indicating a general disruption. A key difference in MiCASA compared to the previous analysis is that MiCASA is an unbiased approach that analyses data from multiple entire sections. The starting point of the analysis is also different; indeed simply generating the whole-section reconstructions from high-resolution images demonstrated previously unappreciated aspects of tDC localization in the wild-type thymus. In the end, the MiCASA result does not so much conflict with the previous conclusion of displacement of tDCs, but rather indicates that the phenotype may be more complex that previously described, and the underlying biological phenotype more profound. These data suggest that XCL1 may play a role not only in DC accumulation within the medulla, but more specifically in the overall organization of tDCs within the medulla, and further suggests that disrupting XCL1 signalling causes changes in medullary organization itself. Further mechanistic studies will be needed to investigate this conclusion.

Our analysis of fetal spinal cord has many differences with the adult thymus samples used in our initial analyses. Most obviously, it is significantly smaller and has fewer cells. In addition, the markers we used for this analysis are exclusively nuclear, resulting in a less complex staining pattern. Finally, some of the markers overlap in a subset of the cells, in contrast to the nonoverlapping patterns we used in our analysis of adult thymus. The main difference between the spinal cord and thymus log-spectra and coherence plots is the width of the lines, reflecting the 99% CI, which are much broader in the fetal spinal cord analysis. This difference may reflect a higher variability in the specific cellular organization in these samples, but is likely strongly influenced by the smaller number of cells in the tissue at this fetal stage. These results suggest that for smaller tissues more subtle differences in organization might be more difficult to detect. The use of much higher *N* values would likely reduce the 99% CI, increasing the discriminatory power of this type of analysis.

In summary, the MiCASA approach to analyse organ structure provides an unbiased, statistically sound method for identifying phenotypes. By using the pairs of markers that mark either the cortex and the medulla CD205/K14 or two medullary markers K14/UEA-1, MiCASA can be used as a standard approach to evaluate corticomedullary organization of the thymus. MiCASA could be particularly useful for temporal analyses, for example, detecting initial stages and progression of cancer, or of thymic involution with aging. As it can be used with any pair of markers, MiCASA can also be used to identify changes in the relationships of specific markers, and act as a hypothesis generator for further mechanistic studies. The current data clearly show the value of MiCASA as a hypothesis generator, indicating novel phenotypes that can be investigated by further mechanistic studies. Because this approach is not based on any unique characteristic of the thymus, it can also be broadly applicable to studies of other tissues and organs.

## Methods

### Mouse strains and genotyping

The *Foxn1*^*LacZ*^ mutants were generated and maintained in the Manley lab on a C57Bl/6J background as previously described^13^. Control mice used in the experiments were littermates of the mutant mice. The *Xcl-1* mutant line was maintained in the Takahama lab on a Balb/c background^14^. All postnatal mice used were males, 2–6 weeks old as indicated in the text and figure legends. Both strains were genotyped by PCR as previously described^13^,^14^. C57Bl/6J mice for colony maintenance were obtained from the Jackson Labs; Balb/c wild-type mice for the *Xcl-1* line were obtained from the Jackson Labs. All experiments involving animals were approved by the Animal Care and Use Committees of the appropriate institution.

### Sample preparation and immunohistochemistry

For adult thymus: Thymi were collected from mice and separated the two lobes, followed by embedding into optimum cutting temperature (OCT) compound (Tissue-Tek), and then snap-frozen on dry ice. Serial frozen sections (10 μm) were air-dried for 30 min before acetone fixation. Thin sections were blocked with normal serum and subsequently incubated with optimal dilutions of primary antibodies (Abs) for at least 1 h at room temperature before washing and incubation with appropriate fluorochrome-conjugated secondary reagents. Controls included slides incubated with non-immune species matched or isotype-matched mouse secondary Abs. For double staining, the sections were incubated simultaneously with primary Abs from different species. The following antibodies were used: anti-β5t (PD021, MLB, 1:200), biotinylated UEA1 (B-1065, Vector Labs, 1:400), anti-CD205 (dp200, Abcam, 1:200), anti-K14 (AF64, Covance, 1:500) and anti-CD11c (HL3, BD Pharmingen, 1:100). Fluorochrome-conjugated anti-Ig secondary reagents were purchased from Jackson ImmunoResearch (West Grove, PA). Alexa Fluor 488 Donkey Anti-Rabbit IgG (711-545-152, 1:800) and Alexa Fluor 594 Donkey Anti-Rabbit IgG (711-585-152, 1:800). Binding of biotinylated Abs was detected by fluorochrome-conjugated streptavidin, Anti-streptavidin Alexa Fluor 488 (S11223, Invitrogen, 1:800).

For fetal spinal cord: Wild-type E15.5 mouse embryos (C3HFeHeJ) were collected, fixed in 4% paraformaldehyde, embedded in paraffin and serially sectioned (6 μm sections). Transverse sections at the level of the forelimb were dewaxed, rehydrated and subjected to antigen retrieval (Na-citrate method). Sections were immunostained with the following antibody combinations: mouse anti-CoupTF-II (R&D Systems, H7147, 1:200), rabbit anti-Pax2 (Covance, PRB-276P, 1:200) and sheep anti-Chx10 (Exalpha, X1179P, 1:200), followed by staining with secondary antibodies, Cy2-conjugated anti-mouse (Cat #715-225-150), Cy3-conjugated donkey anti-sheep (Cag#713-165-147), Cy5-conjugated donkey anti-rabbit (Cat #711-225-152; Jackson ImmunoResearch, 1:800) and 4,6-diamidino-2-phenylindole.

### Imaging and assembly of composite images

Microscopic analysis and digital photography were performed with a Zeiss Axioplan2 microscope (Zeiss, Melville, NY). For each section, tiled, overlapping high-resolution images were acquired using a × 40 objective, and were collected and named in their order of assembly. The degree of overlap required for accurate assembly varies depending on the density of markers, and must be sufficient for accurate and unambiguous matching of individual images during assembly of the final image. Overlapping tiled images were reconstructed into single-image files using the PTGui software package, and manually curated to ensure accurate assembly. For the wild-type sections used for Supplementary Fig. 1, overlapping tiled images were collected and assembled on a Keyence BZ-X700 microscope using their Advanced Acquisition software module. In both cases, the final assembled whole-tissue image should accurately reflect the original section with minimal distortion.

### MiCASA

We use the logarithm of the circularly averaged spectrum (log-spectrum) and arctanh of the circularly averaged cross-coherence (atanh-coherence) to describe cellular structure in the thymus. These quantities are related to spatial correlation functions, but are computed using Fourier transforms in the frequency domain. To compute these quantities, we use multitaper spectral analysis methods. Multitaper methods are robust methods for computing Fourier transforms and related quantities from noisy data and have been used extensively in the estimation of spectral and harmonic content in time series data^11^,^12^,^15^. We call our analysis method MiCASA. In the Supplementary Note, we describe the MiCASA algorithm in detail. MiCASA results in statistical estimates of the log-spectrum, its variance, the atanh-coherence and its variance. It allows the user to determine statistically significant differences between cellular distributions arising from different conditions. The log-spectrum provides information on the cellular distribution of a given cell type, that is, large values of the log-spectrum at frequency *f* indicate that cells are distributed with separations at a characteristic length scale given by the period, 1/*f*. The atanh-coherence provides information on the relationship between two cellular distributions. Large values of the atanh-coherence are indicative of similarities in the distribution of two cell populations as a function of spatial frequency. See the Supplementary Note for an extended description of how to interpret results from a MiCASA analysis.

### Cell quantification using CellProfiler

Sections of XCLKO (*n*=5) and wild-type (*n*=4) mouse thymus were stained with K14 and CD11c antibodies and tiled images taken. Images were then stitched together using PTGui to reconstruct individual sections for analysis. We then used CellProfiler^16^ to identify medulla as the K14-positive region. To identify the CMJ, we used CellProfiler to mask off all cells except the 100 μM region surrounding the medulla. The subcapsular region was defined as the area 100 μM inside the perimeter of each section, and the cortical region was defined as the remaining K14-negative regions (Supplementary Fig. 2). Once each region of the thymus was defined, we determined the total number of dendritic cells within each region using CellProfiler. Total cell counts for medulla, CMJ, cortex and subcapsular region were divided by the total area of each region in order to determine the dendritic cell density.

### Code availability

The MiCASA programme is coded in MATLAB R2014b and can be accessed through a graphical user interface (GUI). Users should note that they also need the open access Chronux package (http://Chronux.org). The user may select multiple experimental conditions for comparison. The GUI takes either RGB or paired single-channel images for each condition. In the case of the single channel, the GUI assumes that both images have the same name, but contain a unique identifier for each image. In the RGB case, it is assumed that the data to be compared are stored in the red and green channels of the image. The MiCASA programme is provided as Supplementary Software. The most current version can also be downloaded at http://research.genetics.uga.edu/MiCASA. This download site also includes the wild-type data sets used to generate the graphs in Supplementary Fig. 1 and links to tutorial videos (https://youtu.be/g63w5bEmXro; https://youtu.be/uHhK5b7nPWk).

### Data availability

All data sets are available on request from the corresponding author. The wild-type data sets used to generate Supplementary Fig. 1 are available on the MiCASA code download site (see Code availability section).

## Additional information

**How to cite this article:** Sornborger, A. *et al*. MiCASA is a new method for quantifying cellular organization. *Nat. Commun.* **8**, 15619 doi: 10.1038/ncomms15619 (2017).

**Publisher's note:** Springer Nature remains neutral with regard to jurisdictional claims in published maps and institutional affiliations.

## Supplementary Material

Supplementary Information Supplementary Figures, Supplementary Note and Supplementary References

Supplementary Software Software for the MiCASA program. The MiCASA program is coded in MATLAB R2014b and can be accessed through a graphical user interface (GUI). Users should note that they also need the open access Chronux package (http://Chronux.org). The GUI takes either RGB or paired single channel images for each condition. In the case of the single channel the GUI assumes that both images have the same name, but contain a unique identifier for each image. In the RGB case it is assumed that the data to be compared is stored in the red and green channels of the image. The most current version of the MiCASA program can also be downloaded at http://research.genetics.uga.edu/MiCASA. This download site also includes the wild-type datasets used to generate the graphs in Supplementary Figure 1 and links to tutorial videos (https://youtu.be/g63w5bEmXro; https://youtu.be/uHhK5b7nPWk).
