# Supplementary material for: MiCASA is a new method for quantifying cellular organization
Source: Nat Commun. 2017 May 30;8:15619. doi: 10.1038/ncomms15619 (PMC5493597; doi:10.1038/ncomms15619)
Supplement: Supplementary Information — Supplementary Figures, Supplementary Note and Supplementary References [file ncomms15619-s1.pdf]

Supplementary Figure 1. MiCASA comparison of medullary organization in two wild-type thymi. The first medullary marker (UEA-1) is green and the second medullary marker (K14) is red. The reconstructed tiles images are shown to the right, with the locations of the high resolution insets indicated with white boxes. Graphical outputs from MiCASA analysis comparing one wild-type thymus to the other are shown on the left, as indicated on the figure: the UEA-1 log-spectrum (green) the K14 log-spectrum (pink), and the atanh-coherence (purple). All MiCASA results are plotted as 99% confidence bands. Scale bar = 150μm.

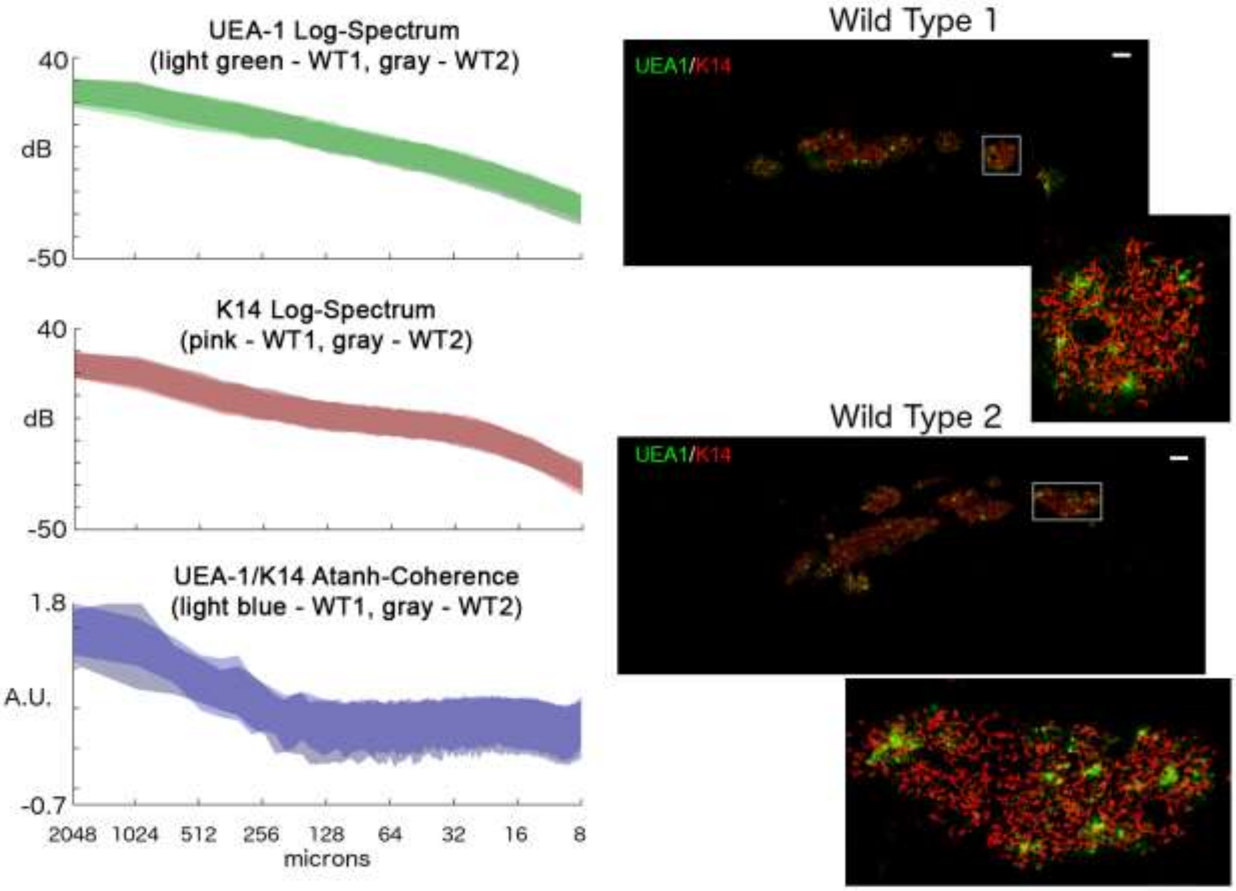

Supplementary Figure 2. Distribution of dendritic cells in Xcl-1 KO and wild-type thymus. **a.** Dual color image of a 6 week old adult male wild-type thymus sagittal section stained for K14 (red) and CD11c (green) from the dataset used in Figure 5. Image shown is reconstructed from multiple high resolution tiled images. (**b-e)** Monochrome single color images from the image shown in **a**. **b.** Masks used for medulla, sub-capsular, cortex and CMJ classified by CellProfiler™. (**c, d, e)** In each pair of panels, the left panel shows the masked CD11c+ single-color image; in the right panels, each dot (shown in different colors) represents one cell as called by CellProfiler™. **c.** cell calls of subcapsular region. **d.** cell calls of cortex. **e.** cell calls of CMJ; in this panel multiple cell calls overlap, giving the appearance of larger individual cells when viewed at low magnification. **f.** Density (# cells per unit area) of CD11c+ cells in sub-capsular region, cortex and CMJ. **g.** Numbers of CD11c+ cells in sub-capsular region, cortex and CMJ. Scale bar = 300μm

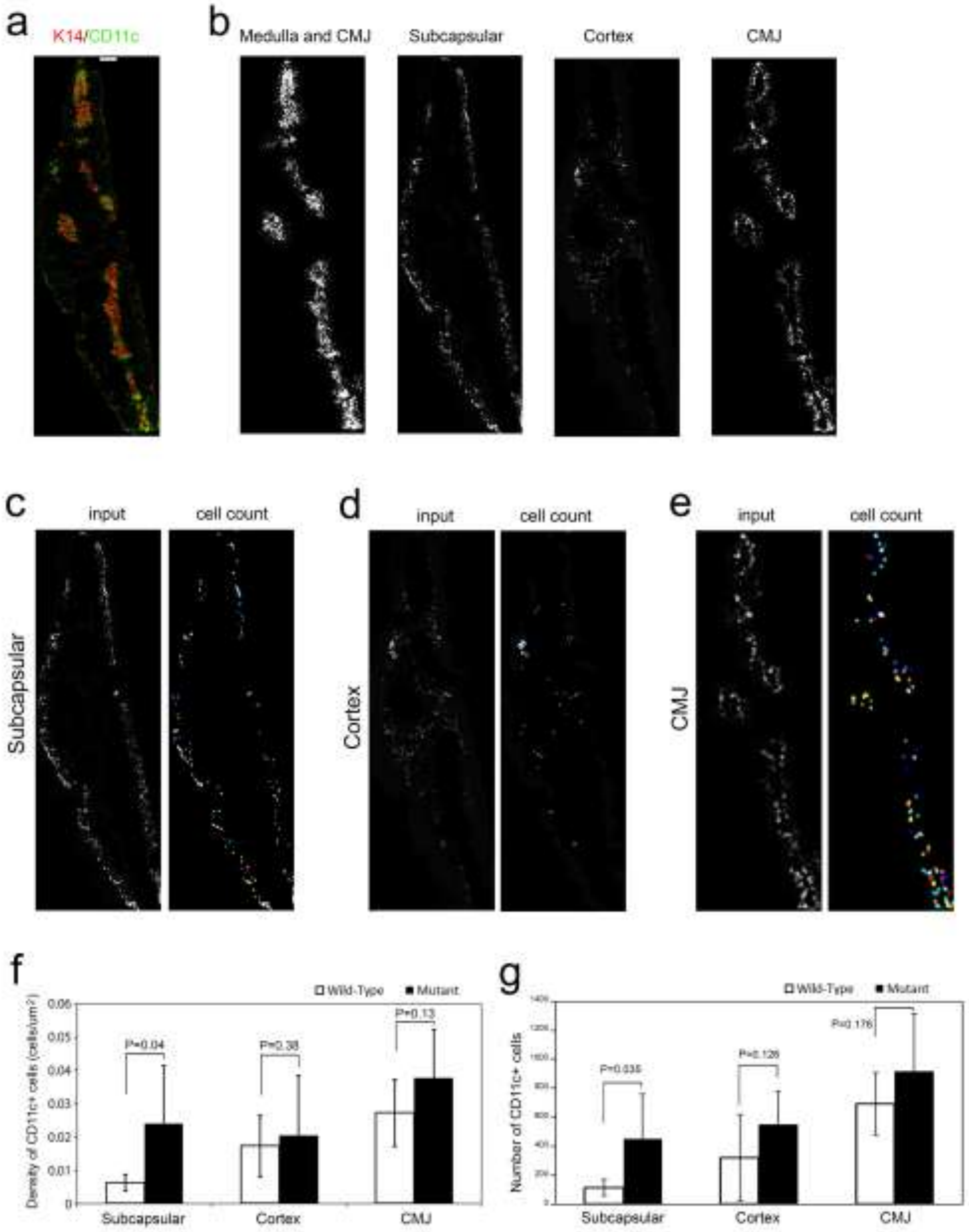

Supplementary Note

MULTITAPER, CIRCULARLY AVERAGED SPECTRUM ANALYSIS (MICASA)

ANDREW T. SORNBORGER, JIE LI, CULLEN TIMMONS, FLORIA LUPU, JONATHAN EGGENSCHWILER, YOUSUKE TAKAHAMA, AND NANCY R. MANLEY

In order to describe structure at various length scales in the thymus, we use the circularly averaged spectrum and circularly averaged cross-coherence. The circularly averaged spectrum is used to describe important features of the distribution of a given cell population at various length scales and the circularly averaged cross-coherence is used to describe similarities between cell populations at various length scales. Since this paper is aimed toward an audience of experimental geneticists and cellular and developmental biologists, we will first describe in some detail the concepts upon which the quantitative analysis is based, then describe the particular techniques that we use to get stable statistical estimates of the quantities of interest.

Convolutions, Frequency Spectra and Coherences

One Dimension. The quantities that we will use to measure structure are based on spatial correlation functions. In one spatial dimension, the autocorrelation function, C(x), is defined by the expression

$$C(x) = \int_{-\infty}^{\infty} f(y-x)f(y)dy \ .$$

Here, $f(x)$ is a function that describes the quantity of interest, say cell density, along a line. The autocorrelation function measures how similar $f(y)$ is to itself when shifted by a distance x, i.e. $f(y-x)$.

An integral of the form

$$(f * g)(x) \equiv \int f(y-x)g(y)dy$$

is called a convolution. Thus, $C(x)$ is the convolution of a function with itself, $C(x) = (f * f)$.

The Fourier transform is important when considering autocorrelation functions (and convolutions in general) for two reasons: 1) the Fast Fourier Transform (FFT) algorithm can be used to rapidly compute convolutions and 2) although the autocorrelation function at point x is typically

statistically correlated with the autocorrelation function at nearby points, it can be decomposed into statistically uncorrelated components via the Fourier transform.

The Fourier transform of a function h is defined

$$\mathcal{F}[h](k) = \int_{-\infty}^{\infty} e^{-2\pi ikx} h(x)dx$$

It is a decomposition of h into sinusoidal functions (recall that $\exp(i\theta) = \cos(\theta) + i\sin(\theta)$). This transform has the property (Parseval's theorem) that the Fourier transform of a convolution is equal to the product of the Fourier transforms of the individual functions being convolved,

$$\mathcal{F}[f * g](k) = \mathcal{F}[f](k) \cdot \mathcal{F}[g](k)$$

Because Fourier transforms may be calculated with the FFT algorithm, which is computationally very efficient, convolutions are more quickly calculated by applying Parseval's theorem. The Fourier transform of the autocorrelation function,

$$\mathcal{F}[C](k) = \mathcal{F}[f * f](k) = |\mathcal{F}[f](k)|^2 \equiv S(k)$$

is called the frequency spectrum where k denotes a spatial frequency in our case. Additionally, if f is a stationary random variable, then $S(k_a)$ is independent of $S(k_b)$ when the spatial frequency $k_a \neq k_b$. This means that it is easier to work analytically with the frequency spectrum than the autocorrelation function because one need not worry about statistical dependencies between nearby frequencies when estimating the spectrum, whereas one does when estimating the autocorrelation. Although stationarity (translational invariance) does not strictly apply to the thymus, where obvious regional boundaries occur, it can still be a good approximation on intermediate length scales on which a cellular pattern can repeat many times.

We use a quantity called the cross-coherence, or simply the coherence, to measure similarities between two functions, f and g. The coherence is defined as a Fourier transform of the convolution of two functions normalized by the power in each function taken separately

$$\chi(k) \equiv \frac{\mathcal{F}[f * g](k)}{|\mathcal{F}[f](k)||\mathcal{F}[g](k)|} = \frac{\mathcal{F}[f](k)^* \mathcal{F}[g](k)}{|\mathcal{F}[f](k)||\mathcal{F}[g](k)|}$$

The magnitude of this function ranges between zero and one. If two functions have similar structure, i.e. if they contain sinusoids with similar phases at a given frequency, the magnitude of

the coherence, $|\chi(k)|$, will be closer to one. Otherwise, if the phases are dissimilar at a given frequency, this function will be closer to zero.

1.2. **Two Dimensions**. In two spatial dimensions, the Fourier transform is defined

$$\mathcal{F}_2[h](k_1,k_2) = \int_{-\infty}^{\infty}\int_{-\infty}^{\infty} e^{2\pi i(k_1x_1+k_2x_2)}h(x_1,x_2)dx_1dx_2$$

Here, the function h is decomposed into sinusoidal waves in a two-dimensional plane. Often, this Fourier transform is rewritten in polar coordinates,

$$\mathcal{F}_2[h] = \mathcal{F}_2[h](k,\theta)$$

where k represents the spatial frequency of the wave and θ the direction perpendicular to the wavefront. In the two-dimensional case, the frequency spectrum is also the Fourier transform of the two-dimensional autocorrelation function,

$$S(k_1,k_2) \equiv \mathcal{F}_2[C](k_1,k_2)$$

The coherence in two-dimensions is similarly defined,

$$\chi(k_1,k_2) \equiv \frac{\mathcal{F}_2[f*g](k_1,k_2)}{|\mathcal{F}_2[f](k_1,k_2)||\mathcal{F}_2[g](k_1,k_2)|}$$

Both of these functions are also often written in polar coordinates, $S(k,\theta)$ and $\chi(k,\theta)$. That is, they may be thought of as describing waves of a given spatial frequency, k, with wavefront perpendicular to a given direction, θ.

A useful one-dimensional summary of $S(k,\theta)$ may be calculated by averaging S across all angles, θ. This is called the *circularly averaged spectrum*,

$$S_c(k) \equiv \int_0^{2\pi} S(k,\theta)d\theta$$

In this paper, we use $S_c(k)$ and its companion, the two-dimensional, circularly averaged coherence, $\chi_c(k)$, to quantify cellular distributions. Broadly speaking, $S_c(k)$ provides information concerning the spatial frequencies (inverse length scales) that make up a cellular distribution and $\chi_c(k)$ provides information concerning similarities between cellular distri-

butions at different spatial frequencies. Because they are one-dimensional quantities, they are easy to plot and represent important quantitative features of cellular distributions at a glance.

It should be remembered that some information about the functions f and g will be lost after we take the circular average. Only if the cellular distribution were isotropic would all the information about the cellular distribution be contained in S_c and χ_c. Nonetheless, these are important diagnostics to examine to identify characteristic length scales in cell populations and investigate similarities between cellular distributions.

Multitaper Estimates of Circularly Averaged Spectra and Coherences

The above discussion concerned the main concepts underlying our reasoning for choosing to quantify cellular distributions using circularly averaged spectra and coherences. Because imaging data is inherently noisy, it is important to choose robust, stable methods for estimating these quantities. One such method is multitaper estimation. It is beyond the scope of this paper to describe in full the theoretical basis for multitaper methods (and tapers in general). However, here we will describe the main issues that arise when estimating Fourier transforms from noisy, discretely sampled, finite data, as are found in imaging measurements of cellular distributions.

One Dimension. First, let's consider what happens when we only have a finite portion, say from $x=-x_0$ to x_0, of a function, $f(x)$, from which to calculate a Fourier transform. In this case, the function $f(x)$, which, in principal, exists from $-\infty$ to ∞ is effectively multiplied by a second

$$\mathrm{rect}(x) = \begin{cases} 1, & |x| > x_0 \\ 0, & |x| < x_0 \end{cases}$$

function $\mathrm{rect}(x)$ defined

Once we take the Fourier transform of this product of functions, we find from Parseval's theorem,

In words, the Fourier transform of the original function f multiplied by the rectangle function, rect, results in a smearing (convolution) of the Fourier transform of f by a new function, sinc, which is the Fourier transform of the rectangle function. The sinc function has a central peak

$$\mathcal{F}[f(x)\mathrm{rect}(x)] = (\mathcal{F}[f] * \mathcal{F}[\mathrm{rect}])(k) = (\mathcal{F}[f] * \mathrm{sinc})(k)$$

with oscillations of decreasing amplitude to either side. Convolution with this function means that original sharp peaks in the spectrum are no longer sharp, their amplitude is decreased, and side lobes, i.e. subsidiary peaks arising to either side of what would have been a sharp peak, form. Even if the spectrum has no sharp peaks, the Fourier transform at a given frequency

contains ‘leakage’, i.e. unwanted contributions from other frequencies. What is done to avoid leakage, to the extent possible, is to multiply f by a new function, whose Fourier transform is as sharply peaked as possible and that has low side lobes. An additional complication arises because data in an image is also discretely sampled, i.e. we only have the value of the function $f(x)$ measured for each pixel, not continuously across the interval.

Slepian and Pollack [1] solved the problem of which discrete functions to use to replace the rect function that minimize the amplitude of side lobes (and hence leakage). They found a set of discrete functions, which are now called Slepian sequences, that have maximal amplitude at frequencies within a specified bandwidth, W, and minimal amplitude in side lobes. Slepian sequences are eigenvectors of a special eigenvalue problem. These eigenvectors are well-concentrated in frequency when they have low side lobes. The eigenvectors are well-concentrated when their eigenvalue is close to 1. The number of well-concentrated eigenvectors, M, depends on both the number of points sampled, X, and the bandwidth, W. When the eigenvectors are ordered in decreasing size of their associated eigenvector, the first $M = 2XW - 3$ are well-concentrated.

Statistical Estimation of the Frequency Spectrum, Multitaper Methods. A final problem in computing a frequency spectrum from imaging data is that the data is noisy. That is, the image that is formed by the camera has noise superimposed on its measurements. Therefore, to get a good estimate of the frequency spectrum, some kind of averaging is needed to smooth the data.

Thomson [2] developed a set of methods for the statistical estimation of spectra and coherences using Slepian sequences called *multitaper methods*. The multitaper spectral estimation method uses M well-concentrated Slepian sequences as tapers to minimize the side lobes arising from an estimate calculated from finite, discrete data. In addition, multitaper methods reduce noise by averaging over a set of spectral estimates calculated with different Slepian tapers.

A multitaper spectral estimate is thus an average over the magnitudes-squared of M Fourier transforms of a tapered function,

$$\hat{S}(k) = \text{avg}\{|\mathcal{F}[f(x)\text{slep}_1(x)](k)|^2, \ldots, |\mathcal{F}[f(x)\text{slep}_M(x)](k)|^2\}$$

where slep_i indicates the i’th Slepian sequence. Written out in series form this becomes

$$\hat{S}(k) = \frac{1}{M}\sum_{m=1}^{M}\left|\sum_{x=1}^{X} f(x)\text{slep}_m(x)e^{-2\pi ikx}\right|^2$$

Here, x is a discrete variable. That is, it takes values on an equally spaced grid of lattice points. For this spectral estimate, because the Slepians sequences are orthogonal, meaning they sample uncorrelated parts of the function, f, the estimates are approximately independent as long as the

estimates are separated in spatial frequency by more than the width of the central peak in the Fourier transform of the Slepian sequence, W . Since the number of points imaged, X , is typically fixed by the microscope, M is largely determined by the bandwidth, W , of the Fourier estimates. By choosing a broad bandwidth (large W and hence large M) the spectral estimates are smoother, have smaller variances and are more stable. For narrow bandwidths (small W and hence small M) the spectral estimates show sharper features, but have larger variances and are less stable. Since the linear dimensions of images are typically fixed, one usually sets W such that there is enough smoothing that estimates are stable, but not so much that important features are smoothed out.

Below, we show in an example how the choice of bandwidth affects spectral features. Note the smaller confidence intervals on the estimate with larger M ($M=15$ versus $M=3$), but note that spectral features are smoothed out more in the same spectral estimate ($XW=11$ versus $XW=3$). The user should perform some exploratory study of their data before choosing a value of M to use. Particularly if there are sharp spectral peaks of interest, a smaller value of M should be used. It should also be noted that, since the total degrees of freedom in a MiCASA analysis are increased with each successive image that is analyzed, if there is excessive variance in spectral estimates due to low bandwidths, it can be overcome by increasing the number of images. For this reason, we typically fix XW to be on the order of 3 − 7, giving 3 − 11 degrees of freedom per image.

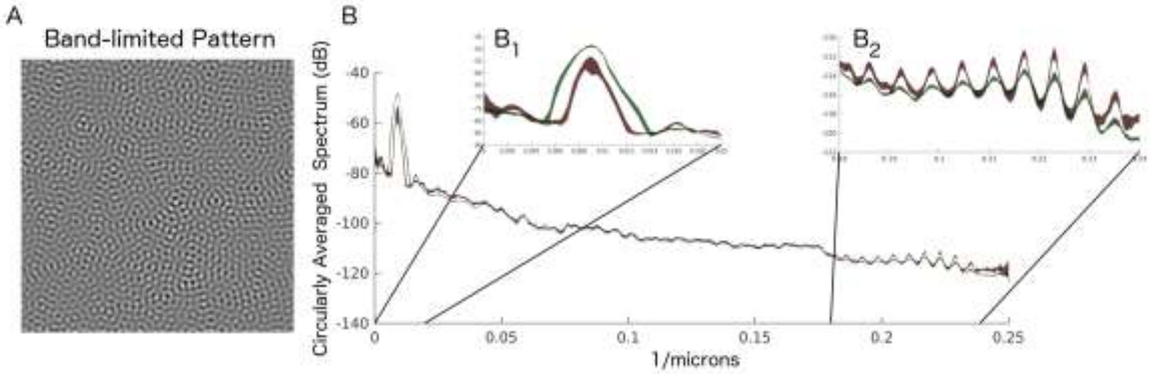

The effect of different bandwidths (W) on spectral estimates. A) A pattern with a sharp ring of spectral power. This is one of N = 10 such images that were used for the MiCASA analysis here. B) A MiCASA spectral estimate of the pattern in (A). $B_{1,2}$) Insets showing details of the MiCASA spectral estimate in (B). Spectral estimate in red (resp. green) indicates XW = 3 (resp. XW = 9); this corresponds to M = 3 (resp. M = 15) degrees of freedom in each spectral of the N = 10 estimates, giving NM = 30 (resp. NM = 150) degrees of freedom in the estimate and an SNR of NM = 5.5 (resp. NM = 12). Note that the red error bars are thicker than the green error bars because the red spectral estimate has fewer (M = 3) degrees of freedom, and therefore a smaller signal-to-noise ratio (SNR). Note, however, that the red spectral estimate has a narrower bandwidth (XW = 3) and therefore sharper spectral features. Inset (B_1) shows the peak due to the wave-like pattern in (A). Note the broadening of the peak in the XW = 9 estimate due to smoothing across a wider bandwidth. Inset (B_2) shows ripples due to (very) low amplitude spectral ripples due to pixelization in (A). Note the smoothing of the ripples in the XW = 9 estimate leading to lower amplitude ripples.

Coherence magnitudes are similarly calculated using a set of Slepian sequences (see MiCASA algorithm, below).

As a last comment, for spectrum S and coherence magnitude, $|\chi|$, $\log(S)$ (log-spectrum) and $\tanh^{-1}(|\chi|)$ (atanh-coherence) are normally distributed, therefore it is these quantities that we will estimate and plot. In addition to this technical consideration, an advantage of plotting log-spectra and atanh-coherence is that statistically significant, low-amplitude features that might be missed in a standard plot, can often be more easily identified.

2.3. **Two Dimensions**. One-dimensional multitaper spectral analysis has been used extensively in signal processing [3]. For the analysis of imaging data, we generalize the method to two dimensions. A Slepian taper is applied along each spatial dimension, giving a two-dimensional Slepian

$$\mathrm{slep}_m(x_1, x_2) = \mathrm{slep}_m(x_1)\mathrm{slep}_m(x_2)$$

The two-dimensional multitaper spectral estimate becomes

$$\hat{S}(k) = \mathrm{avg}\{|\mathcal{F}_2[f(x_1, x_2)\mathrm{slep}_1(x_1, x_2)](k_1, k_2)|^2, \ldots, |\mathcal{F}_2[f(x_1, x_2)\mathrm{slep}_M(x_1, x_2)](k_1, k_2)|^2\}$$

In series notation, this becomes

$$\hat{S}(k_1, k_2) = \frac{1}{M}\sum_{m=1}^{M}\left|\sum_{x_1=1}^{X_1}\sum_{x_2=1}^{X_2} f(x_1, x_2)\mathrm{slep}_m(x_1, x_2)e^{-2\pi i(k_1x_1+k_2x_2)}\right|^2$$

Finally, we circularly average the multitaper spectral estimate

$$\hat{S}_c(k) = \sum_{\theta}\hat{S}(k, \theta)$$

Coherences are handled similarly (see MiCASA algorithm, below).

Jackknife Error Bars. A standard statistical method for determining errors when only a few samples are available or when the exact distribution of a quantity is unknown is the *jackknife* method. This is the method that we use here. One calculates a set of estimates of the quantity of interest, in our case circularly averaged log-spectrum and atanh-coherence, where each member of the set is estimated with one sample left out. An intermediate set of quantities, so-called *pseudo values*, is then computed. This set of estimates has been shown to be effectively normally distributed for most common data sets. Finally, the overall mean and variance of the quantity of interest are calculated. Error bars for a given confidence interval are then obtained from the variance estimates.

The Multitaper Circularly Averaged Spectrum Analysis (MiCASA) Algorithm

Our algorithm is a two-dimensional generalization of a one-dimensional, multitaper spectral analysis described in [2,3]. A typical dataset that we analyze will be comprised of fluorescence measurements at two wavelengths corresponding to fluorophores that label two different cell types, $A(x_1,x_2)$ and $B(x_1,x_2)$. We will have images from a number of experiments, A_n and B_n, where $n=1,\ldots,N$ labels the experiments. To calculate Fourier transforms, we will use $m=1,\ldots,M$ Slepian sequences as tapers. We wish to calculate the circularly averaged spectra and coherences for the two datasets. Combining the concepts and considerations above, we arrive at the following algorithm:

Step 1. Calculate Fourier transforms at both fluorescence wavelengths (i.e. for cell types A and B) for all experiments and tapers,

$$F_A^{i,j} \equiv \mathcal{F}[A^i(x_1,x_2)\mathrm{slep}_j(x_1,x_2)](k_1,k_2)$$

and

$$F_B^{i,j} \equiv \mathcal{F}[B^i(x_1,x_2)\mathrm{slep}_j(x_1,x_2)](k_1,k_2)$$

where $i=1,\ldots,N$ and $j=1,\ldots,M$. (i,j) is then rasterized as a joint index, $p=1,\ldots,NM$, labeling all N experiments and M tapers.

Step 2. Interpolate the Fourier transforms to the same frequency grid. This step is necessary since typically a full 'image' consists of a collage of sub images covering a portion of tissue. Each experiment can correspond to a differently sized image.

Step 3. Compute NM , one-sample-left-out frequency spectrum estimates, $\widehat{S_A^i}$ and $\widehat{S_B^i}$, and coherence magnitudes, $\widehat{\chi_{AB}^i}$, for all experiments and tapers of the two cell types A and B . Here $i=1,\ldots,NM$ labels the left out sample. The spectra are calculated with the expression

$$\hat{S}_X^i = \frac{1}{NM-1}\sum_{p\neq i}|F_X^p(k_1,k_2)|^2$$

where $p=1,\ldots,NM$ and X can be A or B . Here, the sum is multiplied by $1/(NM-1)$ instead of $1/NM$ to give a statistically unbiased estimate. The coherence magnitudes are calculated with the expression

$$\hat{\chi}_{AB}^i = \frac{|\sum_{p\neq i} F_A^{p*}(k_1,k_2)F_B^p(k_1,k_2)|}{\sqrt{\sum_{p\neq i}|F_A^p(k_1,k_2)|^2\sum_{p\neq i}|F_B^p(k_1,k_2)|^2}}$$

Also, calculate $\widehat{S_X^0}$ and $\widehat{\chi_{AB}^0}$, spectra and coherence with no samples left out.

Step 4. Compute

$$\hat{L}_X^i(k) = \int_0^{2\pi} 10\log_{10}(\hat{S}_X^i(k,\theta))d\theta$$

(the circularly averaged log-spectrum in decibels) and

$$\hat{T}_{AB}^i(k) = \int_0^{2\pi} \tanh^{-1}(|\hat{\chi}_{AB}^i(k,\theta)|)d\theta$$

(the circularly averaged atanh-coherence).

Step 5. Compute pseudo values

$$\tilde{L}_X^i = n\hat{L}_X^0 - (n-1)\hat{L}^i$$

and

$$\tilde{T}_{AB}^i = n\hat{T}_{AB}^0 - (n-1)\hat{T}_{AB}^i$$

where $n \equiv NM$.

Step 6. Compute jackknife mean and variance estimates

$$L_X = 1/n\sum_{i=1}^{n}\tilde{L}_X^i$$

$$\sigma_{L_X}^2 = 1/n(n-1)\sum_{i=1}^{n}(\tilde{L}_X^i - L_X)^2$$

and

$$T_{AB} = 1/n\sum_{i=1}^{n}\tilde{T}_{AB}^i$$

$$\sigma_{T_{AB}}^2 = 1/n(n-1)\sum_{i=1}^{n}(\tilde{T}_{AB}^i - T_{AB})^2$$

Compute jackknife confidence intervals for both mean and variance estimates.

Step 7. Plot L_X and T_{AB} with confidence intervals. Plot $\text{Var}(L_X)$ and $\text{Var}(T_{AB})$ with confidence intervals.

Run times for the datasets in this paper took about 12 minutes per image on a 12 core server. It is key to make sure that you have enough RAM for the analysis, otherwise paging to memory can use up a lot of time.

Interpreting a MiCASA Analysis

A MiCASA analysis results in estimates of three circularly averaged functions, $L_A = 10\log_{10}\left(\hat{S}_A\right)$, $L_B = 10\log_{10}\left(\hat{S}_B\right)$ (log-spectra) and $T_{AB} = \tanh^{-1}\left(\hat{\chi}_{AB}\right)$ (atanh-coherence) along with their variances. Units measuring distance, x, will typically be microns and units for spatial frequency, k, will be 2π/microns, respectively. When we plot L_A, L_B and T_{AB}, and their variances, we plot them as a function of $-\log_2(k)$, then label the frequencies in microns (instead of 1/microns). In this way, it is easier to visualize the length-scales of characteristic features of cellular distributions. One disadvantage of this is that the bandwidth of the estimate then varies along the x-axis.

In an analysis of cellular distributions, peaks in the log-spectra at a given frequency indicate extra structure in the cellular distribution, relative to nearby frequencies. If there is a characteristic spatial separation between cells at a distance x, then this will appear as an increase in the log-spectrum at a frequency of $1/x$. Cells characteristically clustered at a given short separation will tend to have a higher amplitude log-spectrum at high frequencies. Clustering in the cellular distribution at long separations will contribute higher amplitudes to the log-spectrum at low frequencies.

Atanh-coherences will have peaks or excess amplitude at spatial frequencies where cells from both cell populations that were imaged have similar separations relative to each other. They will also have peaks if they are anti-correlated with each other (i.e. if one set of cells is absent where the other is present). Note that it is possible to have coherence without high amplitudes in the log-spectra and it is also possible to have no coherence even though both of the log-spectra have high amplitude at a given spatial frequency.

Variances of log-spectra and atanh-coherence are also of interest. When inconsistencies arise between how cells organize, either one population relative to itself (log-spectrum) or one population relative to another (atanh-coherence) in different images or at different locations within a single image, these are measured by variances.

When performing MiCASA on a collage of images, the edges of sub images will tend to generate spurious power in the log-spectra and atanh-coherence. Edges can also increase

variance estimates. This can be minimized by standardizing the original images to have background pixels with zero mean.

Some Details Concerning the Statistical Model

The statistical model governing the MiCASA analysis is the standard repeated measures design. That is, multiple tapered estimates of the spectrum are made on the same image; these estimates are then combined across multiple images to form an aggregate estimate. As was pointed out in [2], since the tapers are orthogonal within the bandwidth of the estimate and very low amplitude outside the bandwidth, the estimates are, to a very good approximation, uncorrelated within each image. Thus, as long as the estimates are independent from image to image, the confidence intervals that we derive are independent between different frequency bands. Note that it is important, in order to assure that estimates are independent from image to image, that the user not use images of multiple tissue slices from the same organ, because nearby slices will very likely be correlated. For the data shown in this paper, the estimates represent NM = 33 and NM = 44 independent degrees of freedom, thus the SNR is high even though the number of images is relatively small.

The hypothesis that estimates are comparable between tapers and images for a given condition may be tested using a fixed-effects, additive, single-factor, repeated measures design using two-way ANOVA. A MiCASA analysis is appropriate if the null hypothesis (i.e. that means across tapers and images are the same) is supported. We note that the hypothesis of interchangeability between tapers and images in our analysis is similar to the ergodic hypothesis in time-series estimates, in which temporal and spatial averages are also checked to be interchangeable.

Supplementary References

[1] Slepian, D. and Pollack, H.O. (1961). Prolate Spheroidal Wave Functions, Fourier Analysis, and Uncertainty - V: The Discrete Case. Bell System Technical Journal, 57, 1371-1430.

[2] Thomson, D.J. (1982). Spectrum Estimation and Harmonic Analysis. Proceedings of the IEEE, 56, 1769-1815.

[3] Mitra, P.P. and Bokil, H. (2008). Observed Brain Dynamics. Oxford University Press. Oxford, UK.
