# Supplementary figures and images for: MiCASA is a new method for quantifying cellular organization
Source: Nat Commun. 2017 May 30;8:15619. doi: 10.1038/ncomms15619 (PMC5493597; doi:10.1038/ncomms15619)

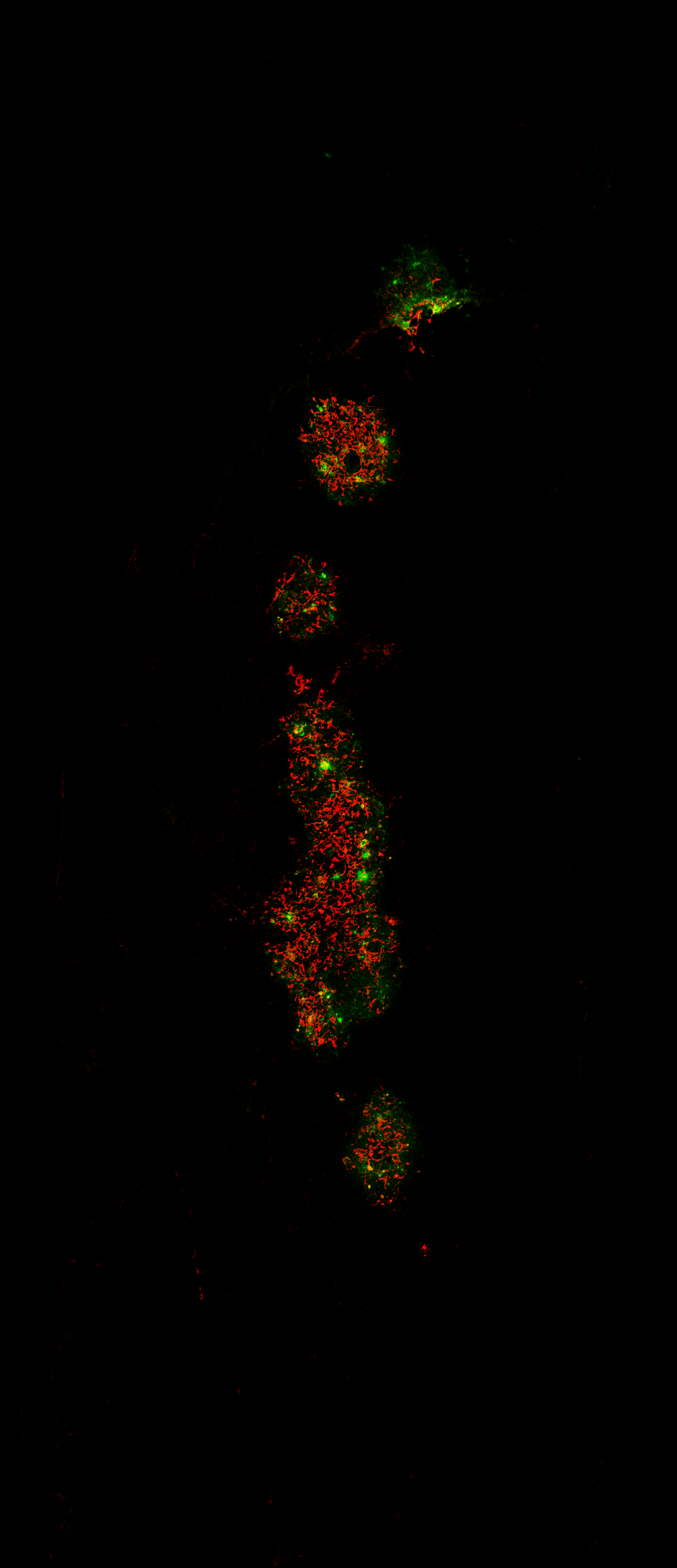

Supplement: Supplementary Software — Software for the MiCASA program. The MiCASA program is coded in MATLAB R2014b and can be accessed through a graphical user interface (GUI). Users should note that they also need the open access Chronux package (http://Chronux.org). The GUI takes either RGB or paired single channel images for each condition. In the case of the single channel the GUI assumes that both images have the same name, but contain a unique identifier for each image. In the RGB case it is assumed that the data to be compared is stored in the red and green channels of the image. The most current version of the MiCASA program can also be downloaded at http://research.genetics.uga.edu/MiCASA. This download site also includes the wild-type datasets used to generate the graphs in Supplementary Figure 1 and links to tutorial videos (https://youtu.be/g63w5bEmXro; https://youtu.be/uHhK5b7nPWk). [file ncomms15619-s2.zip › MiCASA/WildTypeData/WT1-1.tif]

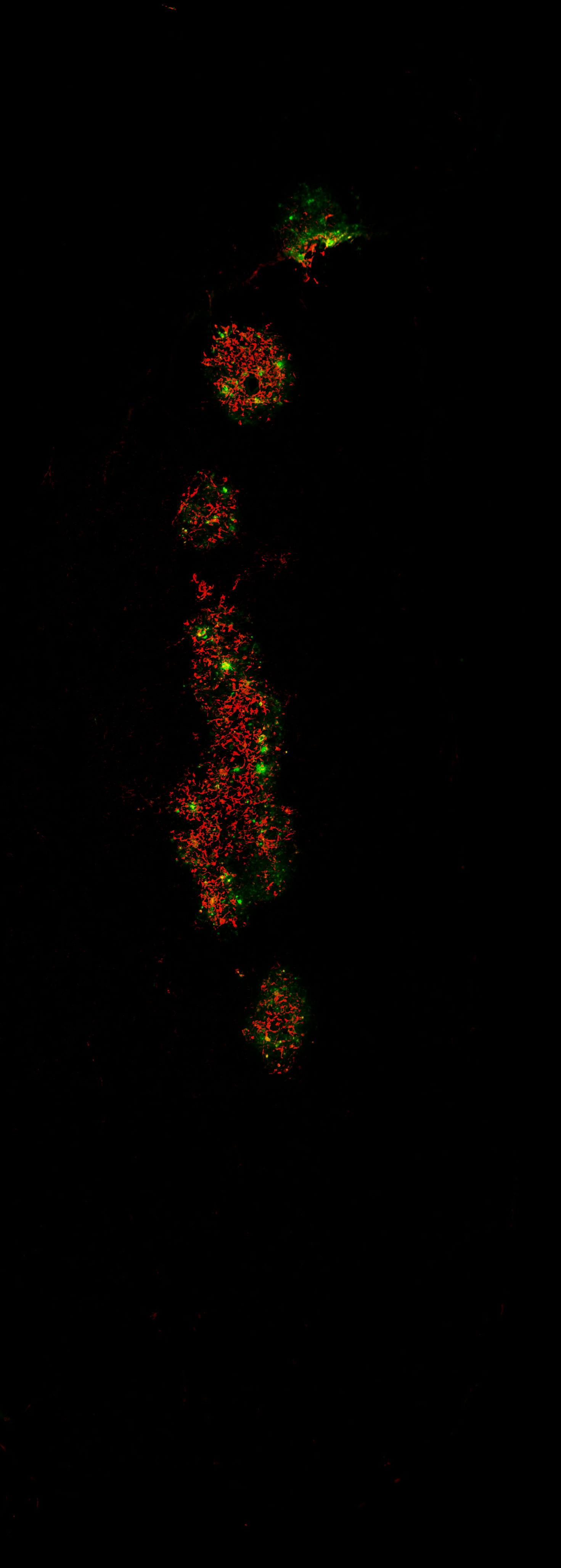

Supplement: Supplementary Software — Software for the MiCASA program. The MiCASA program is coded in MATLAB R2014b and can be accessed through a graphical user interface (GUI). Users should note that they also need the open access Chronux package (http://Chronux.org). The GUI takes either RGB or paired single channel images for each condition. In the case of the single channel the GUI assumes that both images have the same name, but contain a unique identifier for each image. In the RGB case it is assumed that the data to be compared is stored in the red and green channels of the image. The most current version of the MiCASA program can also be downloaded at http://research.genetics.uga.edu/MiCASA. This download site also includes the wild-type datasets used to generate the graphs in Supplementary Figure 1 and links to tutorial videos (https://youtu.be/g63w5bEmXro; https://youtu.be/uHhK5b7nPWk). [file ncomms15619-s2.zip › MiCASA/WildTypeData/WT1-2.tif]

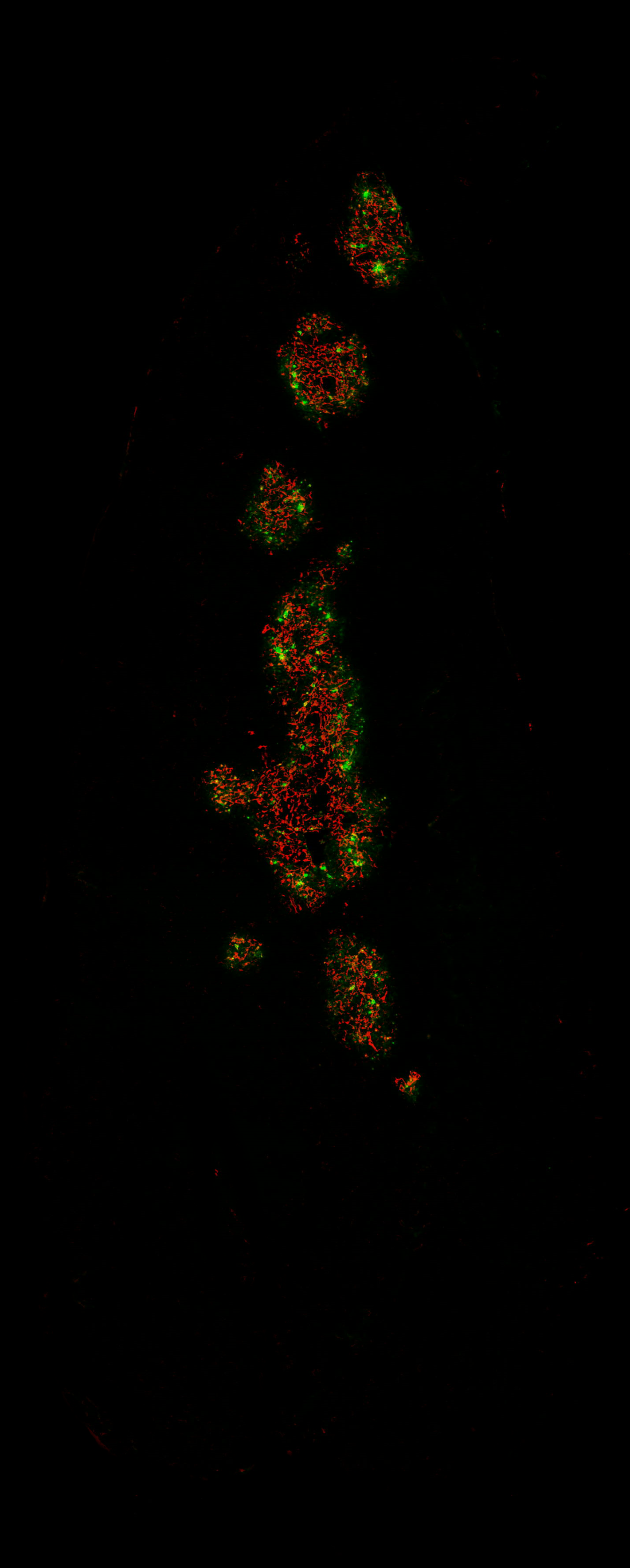

Supplement: Supplementary Software — Software for the MiCASA program. The MiCASA program is coded in MATLAB R2014b and can be accessed through a graphical user interface (GUI). Users should note that they also need the open access Chronux package (http://Chronux.org). The GUI takes either RGB or paired single channel images for each condition. In the case of the single channel the GUI assumes that both images have the same name, but contain a unique identifier for each image. In the RGB case it is assumed that the data to be compared is stored in the red and green channels of the image. The most current version of the MiCASA program can also be downloaded at http://research.genetics.uga.edu/MiCASA. This download site also includes the wild-type datasets used to generate the graphs in Supplementary Figure 1 and links to tutorial videos (https://youtu.be/g63w5bEmXro; https://youtu.be/uHhK5b7nPWk). [file ncomms15619-s2.zip › MiCASA/WildTypeData/WT1-3.tif]

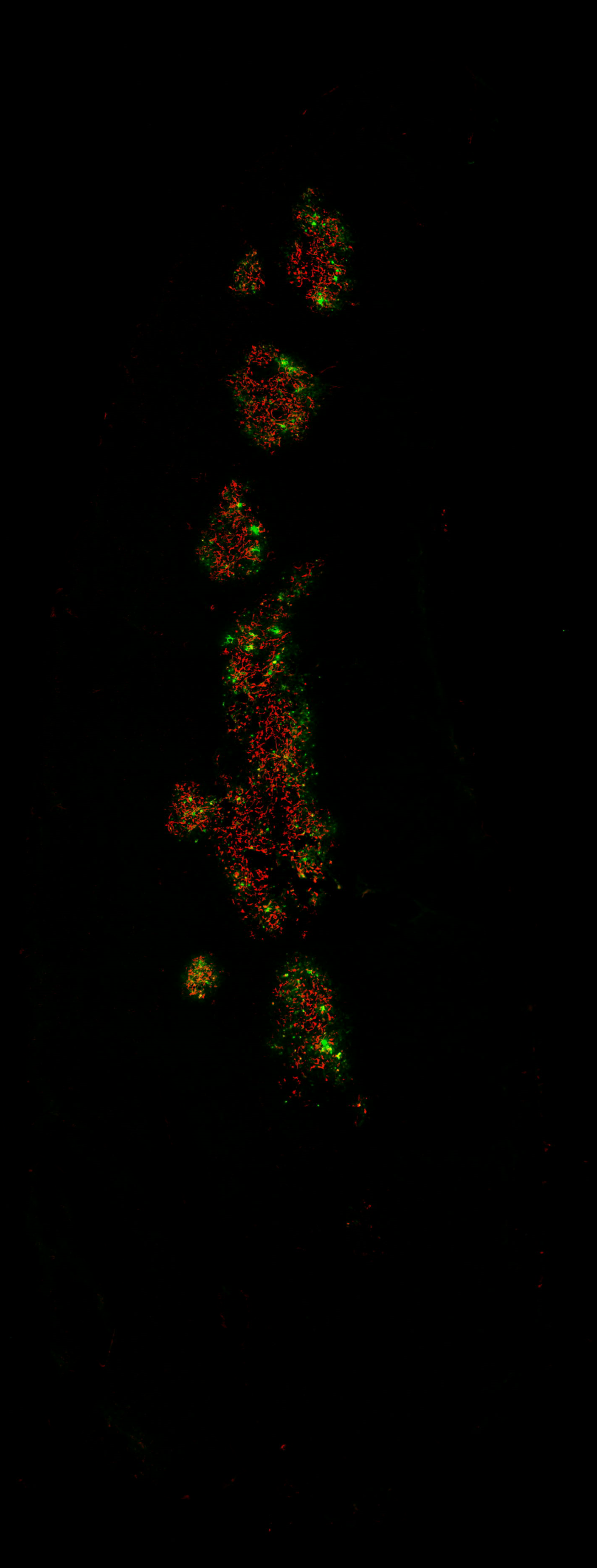

Supplement: Supplementary Software — Software for the MiCASA program. The MiCASA program is coded in MATLAB R2014b and can be accessed through a graphical user interface (GUI). Users should note that they also need the open access Chronux package (http://Chronux.org). The GUI takes either RGB or paired single channel images for each condition. In the case of the single channel the GUI assumes that both images have the same name, but contain a unique identifier for each image. In the RGB case it is assumed that the data to be compared is stored in the red and green channels of the image. The most current version of the MiCASA program can also be downloaded at http://research.genetics.uga.edu/MiCASA. This download site also includes the wild-type datasets used to generate the graphs in Supplementary Figure 1 and links to tutorial videos (https://youtu.be/g63w5bEmXro; https://youtu.be/uHhK5b7nPWk). [file ncomms15619-s2.zip › MiCASA/WildTypeData/WT1-4.tif]

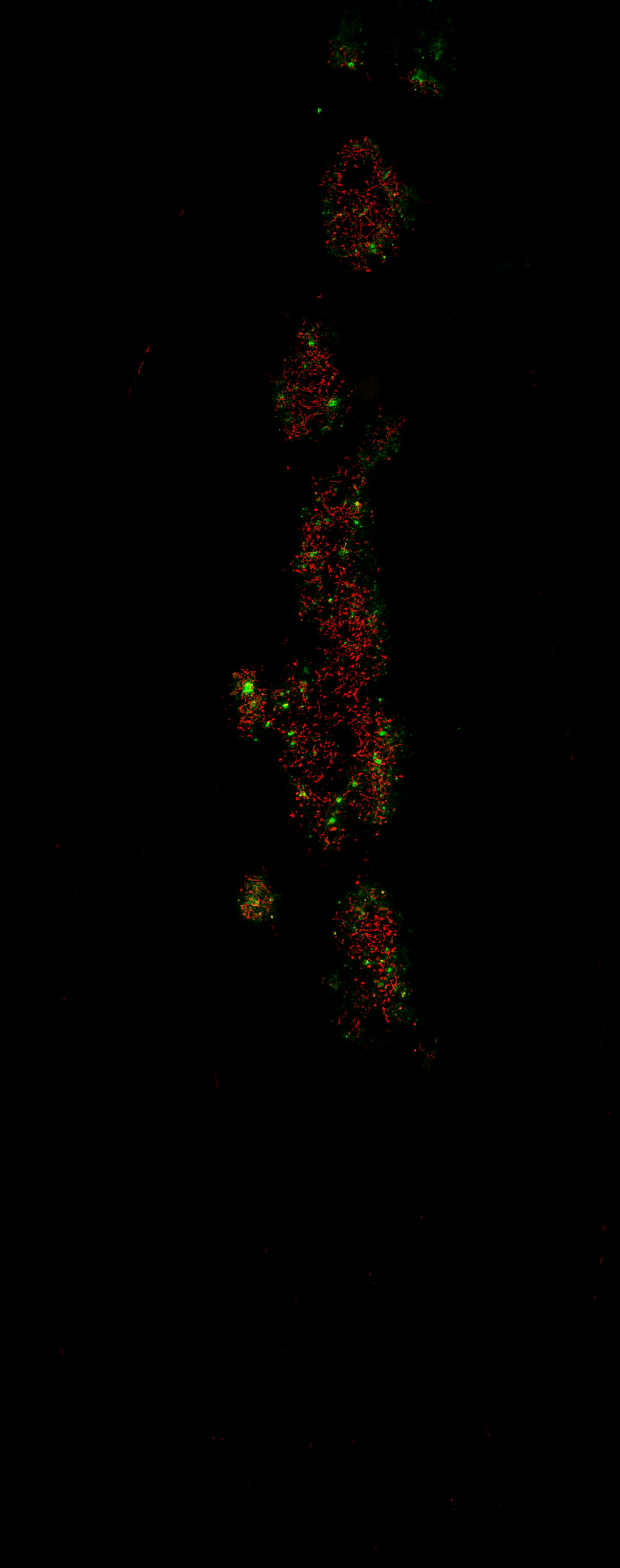

Supplement: Supplementary Software — Software for the MiCASA program. The MiCASA program is coded in MATLAB R2014b and can be accessed through a graphical user interface (GUI). Users should note that they also need the open access Chronux package (http://Chronux.org). The GUI takes either RGB or paired single channel images for each condition. In the case of the single channel the GUI assumes that both images have the same name, but contain a unique identifier for each image. In the RGB case it is assumed that the data to be compared is stored in the red and green channels of the image. The most current version of the MiCASA program can also be downloaded at http://research.genetics.uga.edu/MiCASA. This download site also includes the wild-type datasets used to generate the graphs in Supplementary Figure 1 and links to tutorial videos (https://youtu.be/g63w5bEmXro; https://youtu.be/uHhK5b7nPWk). [file ncomms15619-s2.zip › MiCASA/WildTypeData/WT1-5.tif]

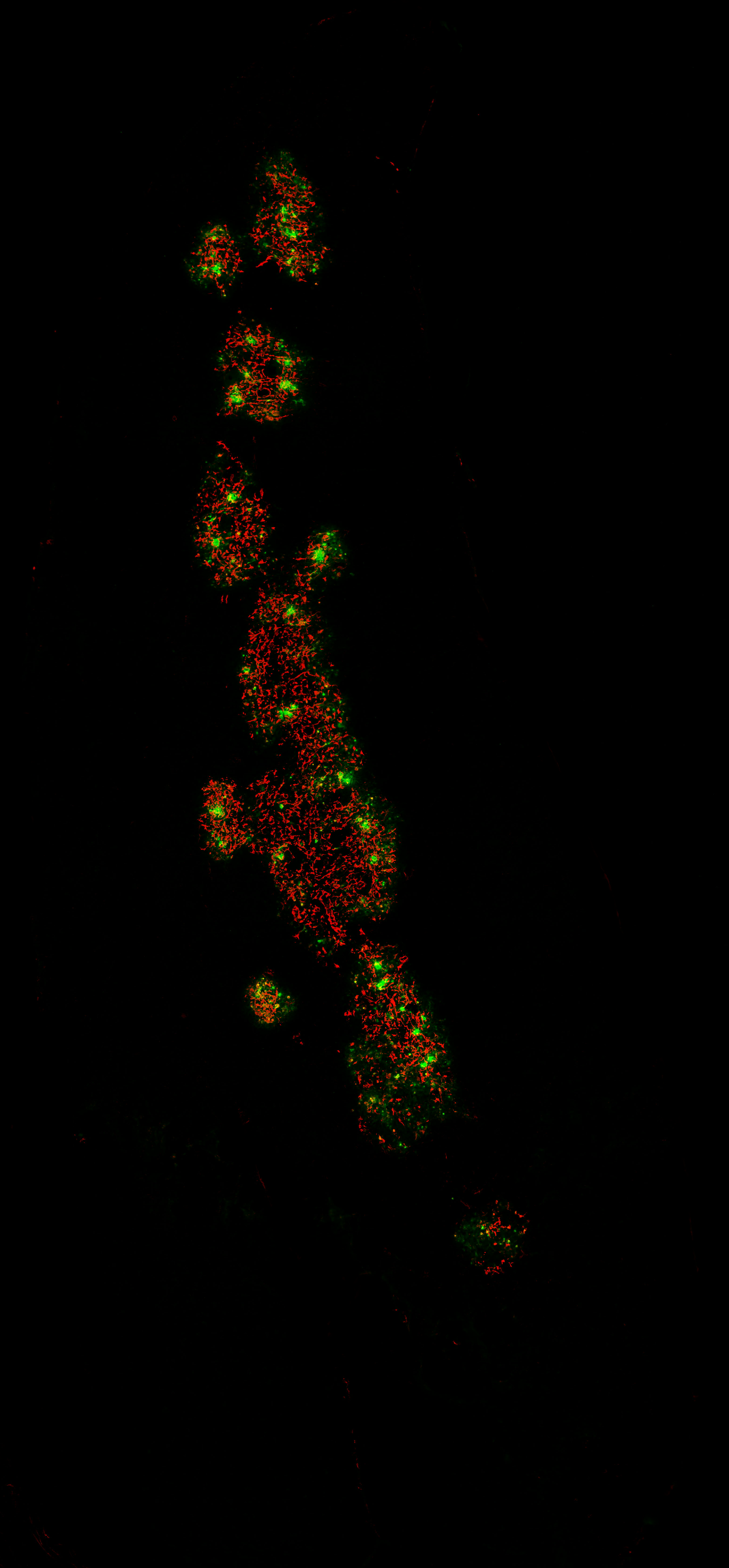

Supplement: Supplementary Software — Software for the MiCASA program. The MiCASA program is coded in MATLAB R2014b and can be accessed through a graphical user interface (GUI). Users should note that they also need the open access Chronux package (http://Chronux.org). The GUI takes either RGB or paired single channel images for each condition. In the case of the single channel the GUI assumes that both images have the same name, but contain a unique identifier for each image. In the RGB case it is assumed that the data to be compared is stored in the red and green channels of the image. The most current version of the MiCASA program can also be downloaded at http://research.genetics.uga.edu/MiCASA. This download site also includes the wild-type datasets used to generate the graphs in Supplementary Figure 1 and links to tutorial videos (https://youtu.be/g63w5bEmXro; https://youtu.be/uHhK5b7nPWk). [file ncomms15619-s2.zip › MiCASA/WildTypeData/WT1-6.tif]

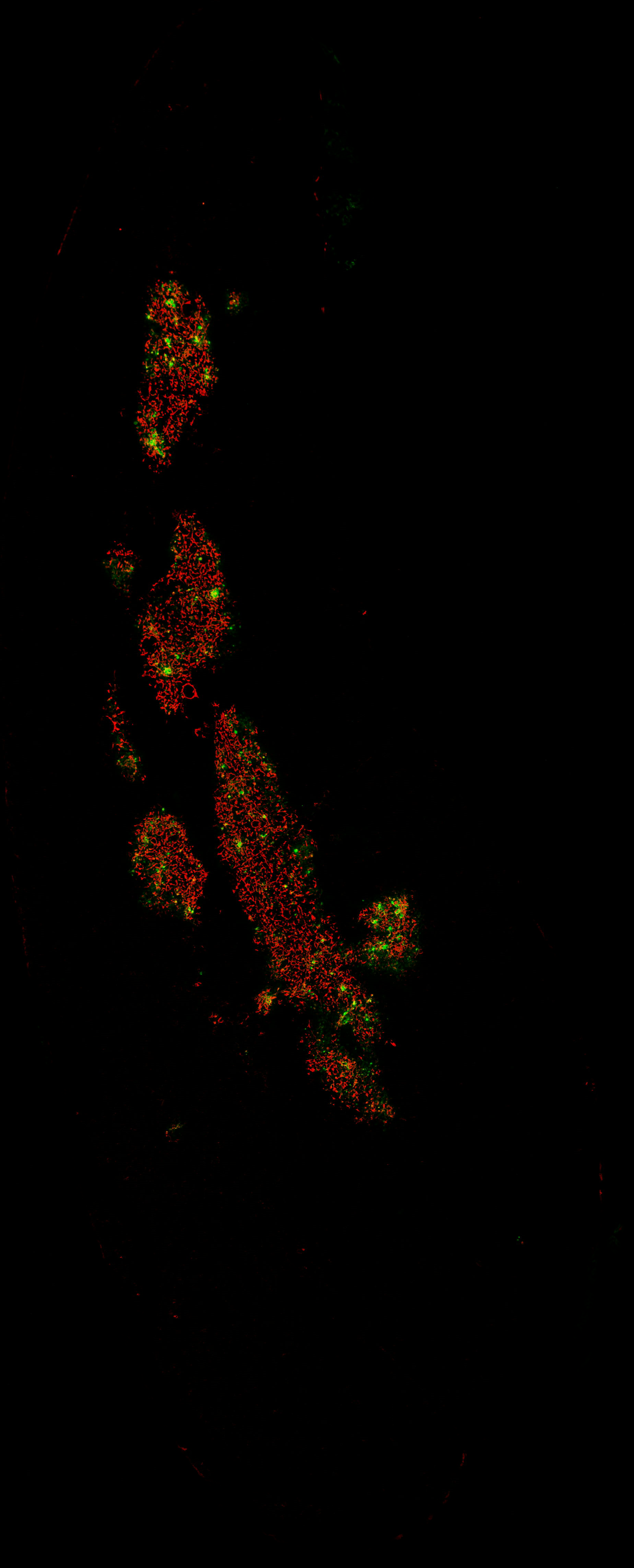

Supplement: Supplementary Software — Software for the MiCASA program. The MiCASA program is coded in MATLAB R2014b and can be accessed through a graphical user interface (GUI). Users should note that they also need the open access Chronux package (http://Chronux.org). The GUI takes either RGB or paired single channel images for each condition. In the case of the single channel the GUI assumes that both images have the same name, but contain a unique identifier for each image. In the RGB case it is assumed that the data to be compared is stored in the red and green channels of the image. The most current version of the MiCASA program can also be downloaded at http://research.genetics.uga.edu/MiCASA. This download site also includes the wild-type datasets used to generate the graphs in Supplementary Figure 1 and links to tutorial videos (https://youtu.be/g63w5bEmXro; https://youtu.be/uHhK5b7nPWk). [file ncomms15619-s2.zip › MiCASA/WildTypeData/WT2-1.tif]

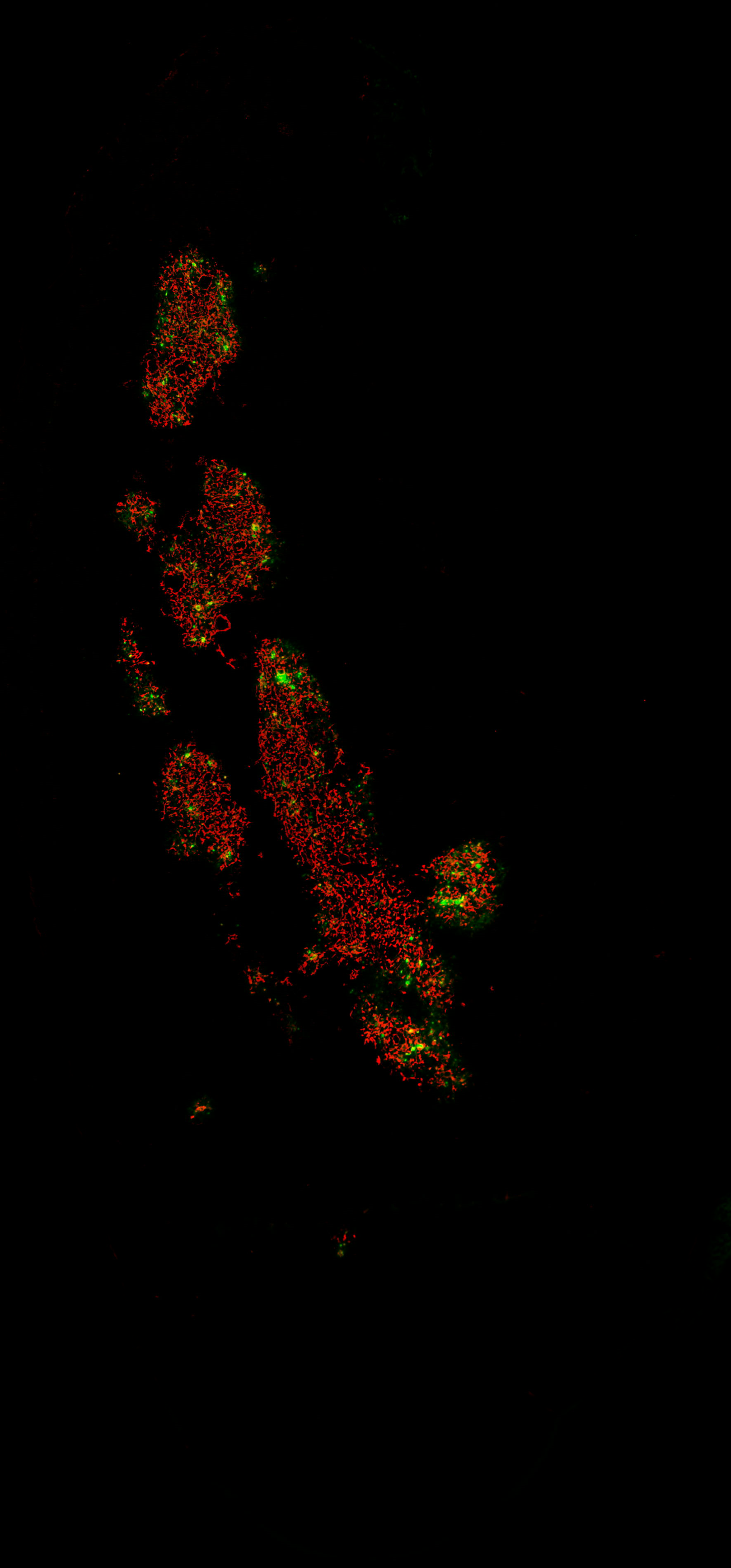

Supplement: Supplementary Software — Software for the MiCASA program. The MiCASA program is coded in MATLAB R2014b and can be accessed through a graphical user interface (GUI). Users should note that they also need the open access Chronux package (http://Chronux.org). The GUI takes either RGB or paired single channel images for each condition. In the case of the single channel the GUI assumes that both images have the same name, but contain a unique identifier for each image. In the RGB case it is assumed that the data to be compared is stored in the red and green channels of the image. The most current version of the MiCASA program can also be downloaded at http://research.genetics.uga.edu/MiCASA. This download site also includes the wild-type datasets used to generate the graphs in Supplementary Figure 1 and links to tutorial videos (https://youtu.be/g63w5bEmXro; https://youtu.be/uHhK5b7nPWk). [file ncomms15619-s2.zip › MiCASA/WildTypeData/WT2-2.tif]

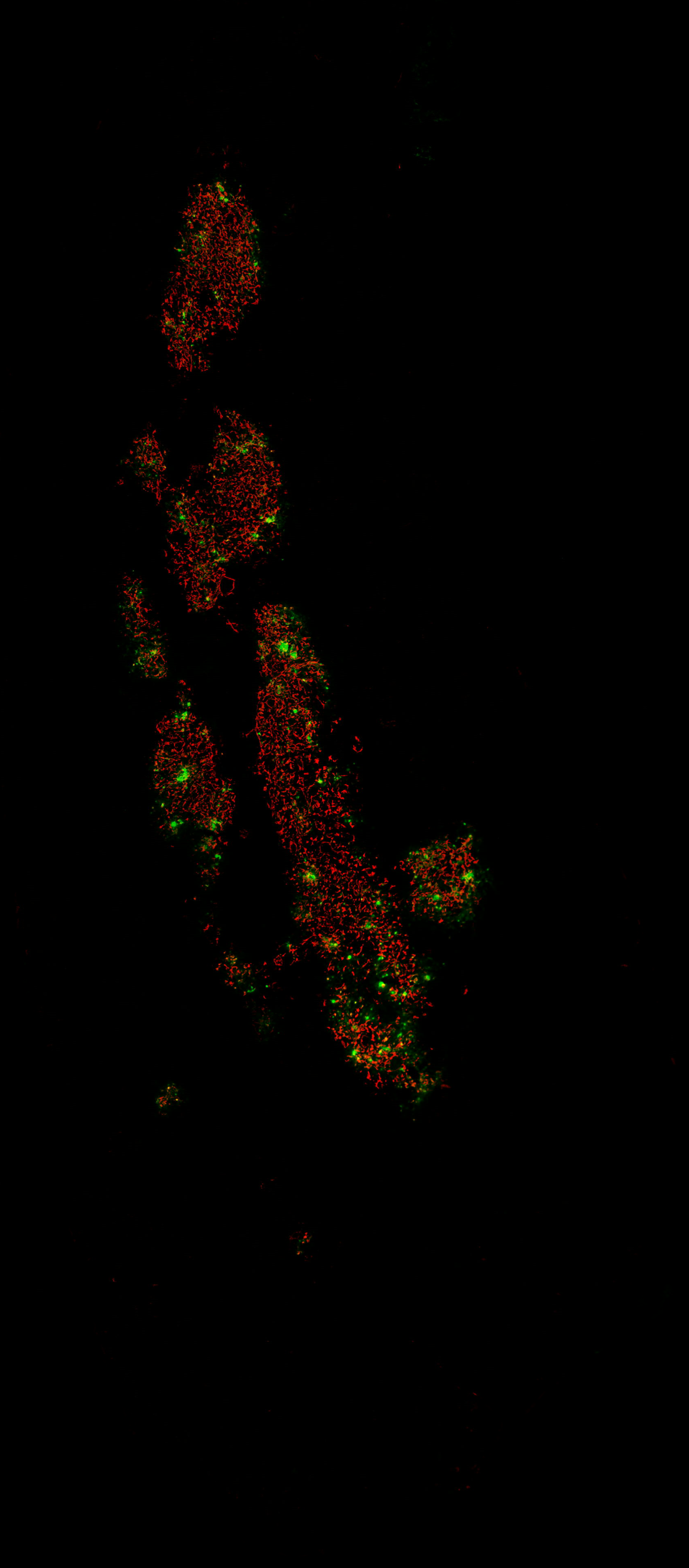

Supplement: Supplementary Software — Software for the MiCASA program. The MiCASA program is coded in MATLAB R2014b and can be accessed through a graphical user interface (GUI). Users should note that they also need the open access Chronux package (http://Chronux.org). The GUI takes either RGB or paired single channel images for each condition. In the case of the single channel the GUI assumes that both images have the same name, but contain a unique identifier for each image. In the RGB case it is assumed that the data to be compared is stored in the red and green channels of the image. The most current version of the MiCASA program can also be downloaded at http://research.genetics.uga.edu/MiCASA. This download site also includes the wild-type datasets used to generate the graphs in Supplementary Figure 1 and links to tutorial videos (https://youtu.be/g63w5bEmXro; https://youtu.be/uHhK5b7nPWk). [file ncomms15619-s2.zip › MiCASA/WildTypeData/WT2-3.tif]

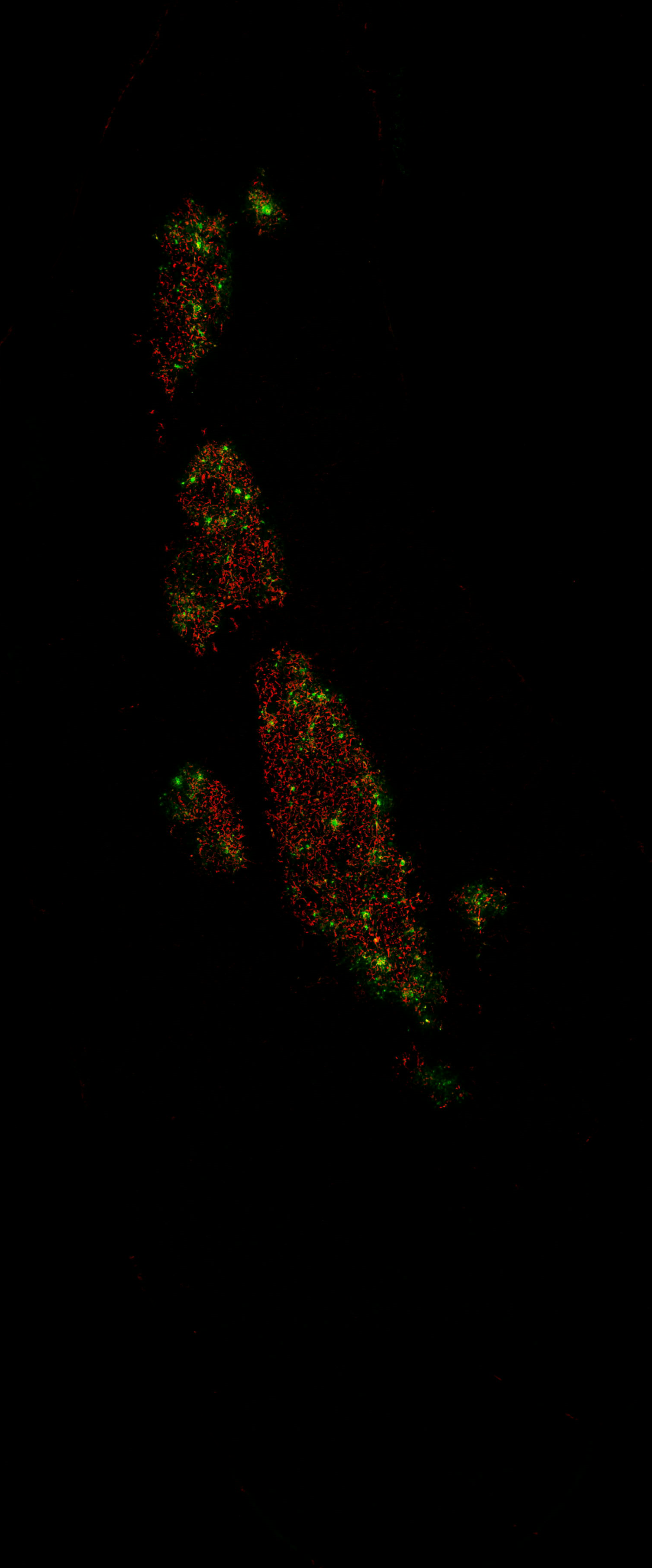

Supplement: Supplementary Software — Software for the MiCASA program. The MiCASA program is coded in MATLAB R2014b and can be accessed through a graphical user interface (GUI). Users should note that they also need the open access Chronux package (http://Chronux.org). The GUI takes either RGB or paired single channel images for each condition. In the case of the single channel the GUI assumes that both images have the same name, but contain a unique identifier for each image. In the RGB case it is assumed that the data to be compared is stored in the red and green channels of the image. The most current version of the MiCASA program can also be downloaded at http://research.genetics.uga.edu/MiCASA. This download site also includes the wild-type datasets used to generate the graphs in Supplementary Figure 1 and links to tutorial videos (https://youtu.be/g63w5bEmXro; https://youtu.be/uHhK5b7nPWk). [file ncomms15619-s2.zip › MiCASA/WildTypeData/WT2-4.tif]

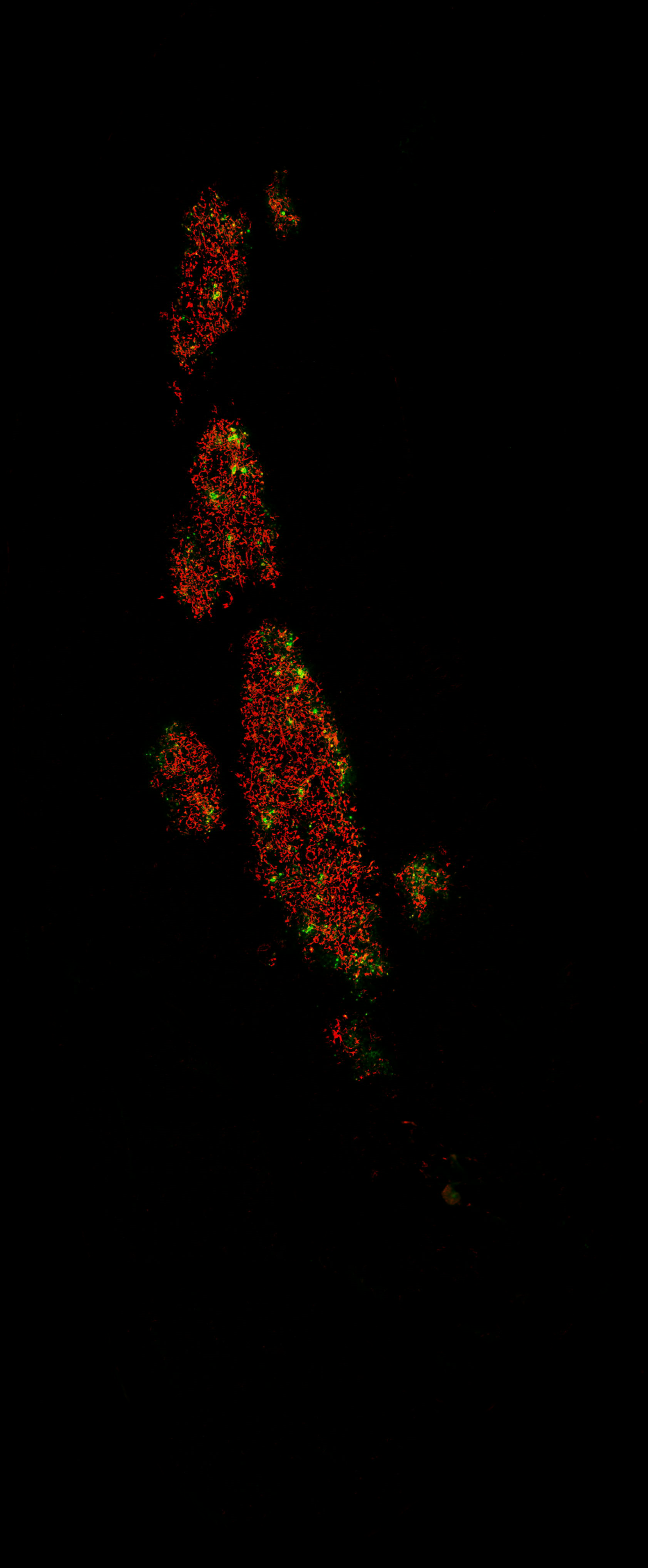

Supplement: Supplementary Software — Software for the MiCASA program. The MiCASA program is coded in MATLAB R2014b and can be accessed through a graphical user interface (GUI). Users should note that they also need the open access Chronux package (http://Chronux.org). The GUI takes either RGB or paired single channel images for each condition. In the case of the single channel the GUI assumes that both images have the same name, but contain a unique identifier for each image. In the RGB case it is assumed that the data to be compared is stored in the red and green channels of the image. The most current version of the MiCASA program can also be downloaded at http://research.genetics.uga.edu/MiCASA. This download site also includes the wild-type datasets used to generate the graphs in Supplementary Figure 1 and links to tutorial videos (https://youtu.be/g63w5bEmXro; https://youtu.be/uHhK5b7nPWk). [file ncomms15619-s2.zip › MiCASA/WildTypeData/WT2-5.tif]

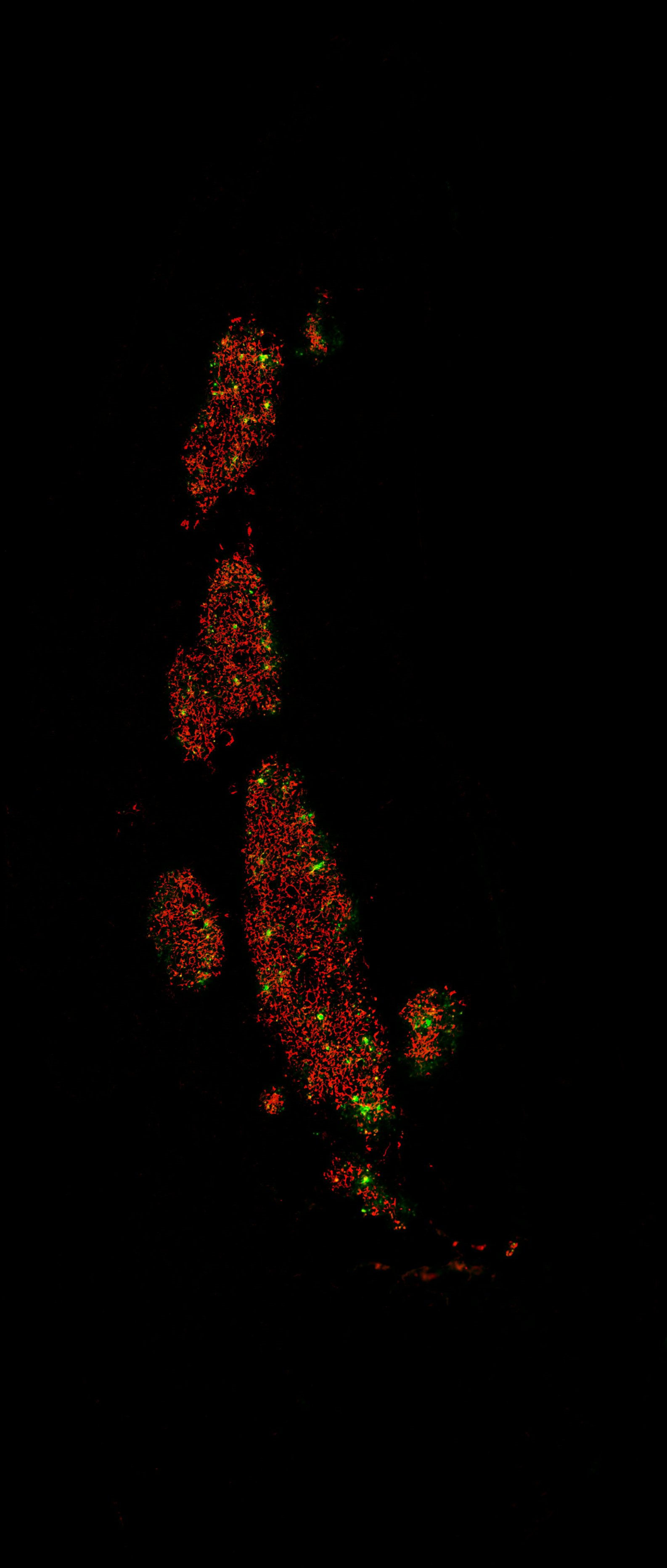

Supplement: Supplementary Software — Software for the MiCASA program. The MiCASA program is coded in MATLAB R2014b and can be accessed through a graphical user interface (GUI). Users should note that they also need the open access Chronux package (http://Chronux.org). The GUI takes either RGB or paired single channel images for each condition. In the case of the single channel the GUI assumes that both images have the same name, but contain a unique identifier for each image. In the RGB case it is assumed that the data to be compared is stored in the red and green channels of the image. The most current version of the MiCASA program can also be downloaded at http://research.genetics.uga.edu/MiCASA. This download site also includes the wild-type datasets used to generate the graphs in Supplementary Figure 1 and links to tutorial videos (https://youtu.be/g63w5bEmXro; https://youtu.be/uHhK5b7nPWk). [file ncomms15619-s2.zip › MiCASA/WildTypeData/WT2-6.tif]
